# Spectroscopy of Wigner crystal polarons in an atomically thin semiconductor

**DOI:** 10.1038/s41567-026-03395-0

**Published:** 2026-08-11

**Authors:** L. Wang, F. Menzel, F. Pichler, P. Knüppel, K. Watanabe, T. Taniguchi, M. Knap, T. Smoleński

**Affiliations:** 1 Department of Physics, University of Basel, Basel, Switzerland; 2 Technical University of Munich, TUM School of Natural Sciences, Physics Department, Garching, Germany; 3 Munich Center for Quantum Science and Technology (MCQST), München, Germany; 4 Research Center for Electronic and Optical Materials, National Institute for Materials Science, Tsukuba, Japan; 5 Research Center for Materials Nanoarchitectonics, National Institute for Materials Science, Tsukuba, Japan

**Keywords:** Quantum fluids and solids, Electronic properties and materials, Phase transitions and critical phenomena, Optical spectroscopy, Two-dimensional materials

## Abstract

Strongly interacting electrons in two-dimensional systems can spontaneously break translational symmetry, forming a periodic Wigner crystal. Although these crystals have been realized in several platforms, experimental studies of their collective many-body excitations in the absence of a magnetic field have not been demonstrated. Here we access this regime optically by uncovering Wigner crystal polarons, which are hybrid light–matter quasiparticles that arise from the dressing of excitons by the collective excitations of the Wigner crystal. These polarons manifest as optical resonances in the cryogenic reflectance spectra of a charge-tunable WSe_2_ monolayer, appearing concurrently with previously identified exciton umklapp transitions. In contrast to the latter, the energies of Wigner crystal polarons are governed not only by the electronic lattice constant but also by their hybridization with attractive exciton–polarons, whose strength is controlled by electronic interactions. These many-body excitations provide an optical interface to the spin state of the Wigner crystal that, as we demonstrate, can be controlled both magnetically and optically. Our work establishes layered materials as a platform for exploring dynamical impurity dressing by strongly correlated electronic orders.

## Abstract (web summary)

Electron Wigner crystals have been observed in atomically thin transition metal dichalcogenides, but their collective excitations have not been shown. Now the signatures of these excitations are revealed in the optical spectra of a charge-tunable monolayer semiconductor.

## Main

Understanding the ground states and collective excitations of strongly correlated electronic phases lies at the heart of condensed-matter physics. A paradigmatic example of such a phase is the Wigner crystal (WC), which forms when Coulomb interactions dominate over kinetic energy in a two-dimensional (2D) electron system^1^. Following studies in conventional semiconductors in which WCs are typically characterized via transport properties^2–7^, interest in WCs has been recently reignited by the advent of van der Waals (vdW) heterostructures based on transition metal dichalcogenide monolayers. With their gate tunability, weak dielectric screening and flat electronic bands, these structures not only provide a tunable platform for realizing WCs^8–16^ but also uniquely enable probing them with excitons—fundamental optical excitations that act as robust bosonic impurities in a fermionic environment^17^. In particular, exciton scattering off the periodic repulsive potential generated by the WC gives rise to an umklapp resonance whose energy serves as a quantitative probe of the WC lattice constant^8,18^. Yet, the opposite regime—where the excitons strongly couple to collective excitations of the WC—has remained elusive so far. This is the realm of polarons: quasiparticles arising from dressing an impurity with excitations of a surrounding medium^17,19–21^, originally introduced to describe electrons interacting with phonons in real crystals^22^. Although recent experiments have begun to explore how correlated phases affect exciton–polarons, particularly in the presence of moiré potentials^23–26^, the nature of polaron dressing by collective excitations in spontaneously ordered electronic crystals—and the possible emergence of hybrid quasiparticles—remains unresolved.

Here we report the direct observation of WC polarons (WPs): light–matter excitations in the resonant optical response of a WSe_2_ monolayer hosting a robust WC at low temperature *T* and electron density *n*_e_. We show that WPs manifest as umklapp replicas of attractive exciton–polarons (APs) and appear within the same *T*−*n*_e_ phase space as the exciton umklapp resonance. In stark contrast to the latter, both WPs exhibit a sizable blueshift relative to the corresponding APs even in the low-density limit, indicating that they arise not only from the Bragg scattering of mobile excitons but also from their dressing by collective excitations of the WC. We show that the intensities of WPs are determined by the spin state of the WC, which we control not only with an external magnetic field but also optically using circularly polarized light. Our results demonstrate the potential of vdW materials for characterizing emergent quasiparticle excitations arising from strong interactions between excitons and charge-ordered electronic phases.

**Fig. 1:**
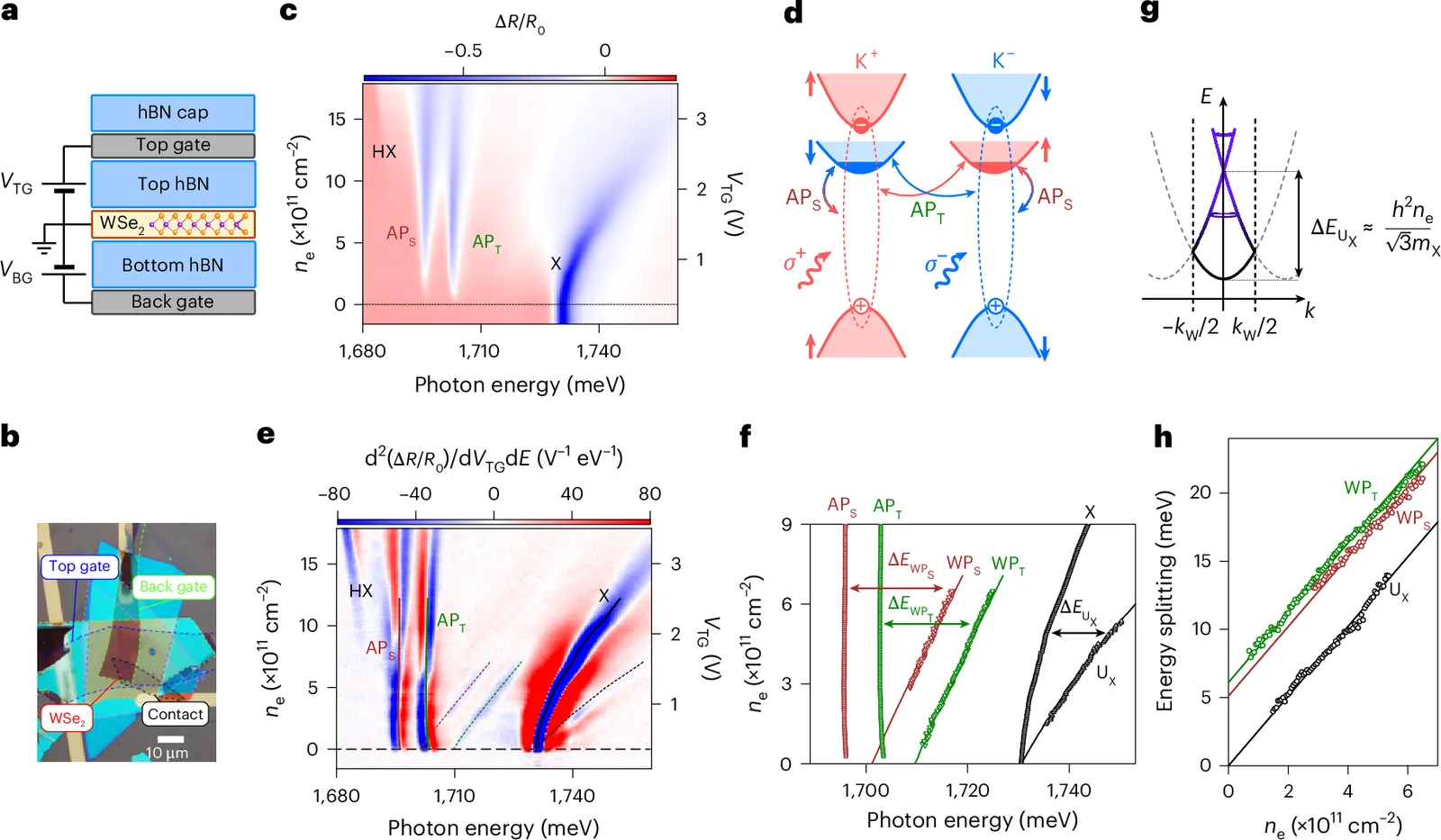
Optical signatures of a WC in a WSe_2_ monolayer. **a**,**b**, Schematic (**a**) and optical micrograph (**b**) of the charge-tunable WSe_2_ monolayer device explored in the main text (see the ‘Device fabrication’ section). **c**, Reflectance contrast spectrum as a function of *n*_e_ at *B* = 0 and *T* = 1.6 K. **d**, Schematic illustrating the band structure of a WSe_2_ monolayer as well as valley-selective optical selection rules. **e**, *n*_e_ evolution of the reflectance contrast differentiated with respect to the gate voltage and photon energy. The solid lines represent the fitted energies of exciton and both AP transitions, whereas the dashed lines mark the corresponding umklapp and WP resonances originating from scattering off the WC. **f**, Energies of X, AP_T_ and AP_S_ transitions, as well as the corresponding umklapp and WP peaks extracted from the data in **c** and **e** in the low-*n*_e_ limit (see the ‘Fitting the spectral energies of optical resonances’ section). **g**, Illustration of the backfolded exciton band structure, giving rise to a blueshifted umklapp resonance (purple). **h**, *n*_e_-dependent energy splittings between each main peak and the corresponding umklapp transition. Each dataset is fitted with a linear dependence $\Delta E={h}^{2}{n}_{{\rm{e}}}/\sqrt{3}{m}_{{\rm{X}}}+\it\varDelta$ with fixed *m*_X_ ≈ 0.68*m*_0_ and different offset: *Δ* = 0 for U_X_, *Δ* ≈ 6 meV for WP_T_ and *Δ* ≈ 5 meV for WP_S_. Source data

**Extended Data Fig. 1:**
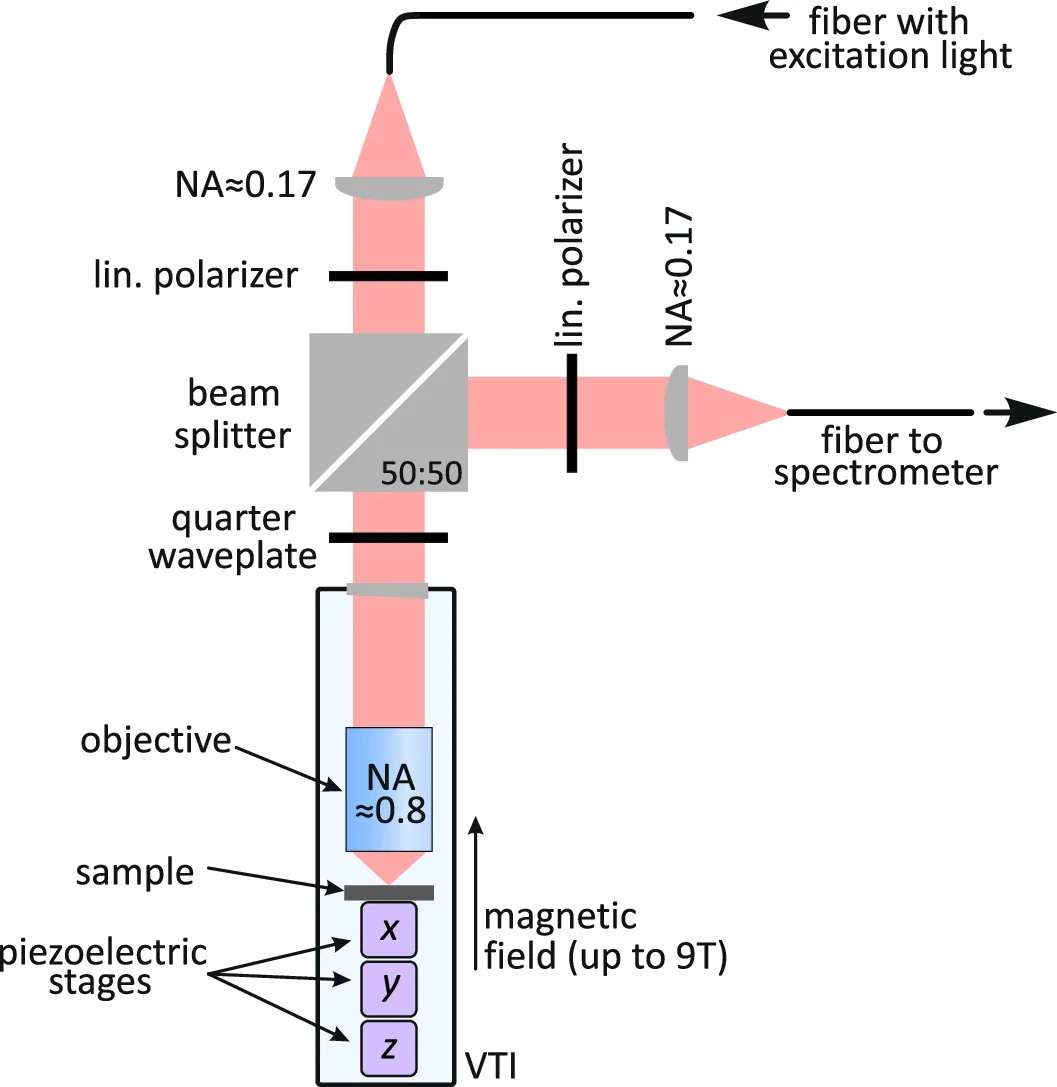
Experimental setup. Simplified schematic of the experimental setup.

**Extended Data Fig. 2:**
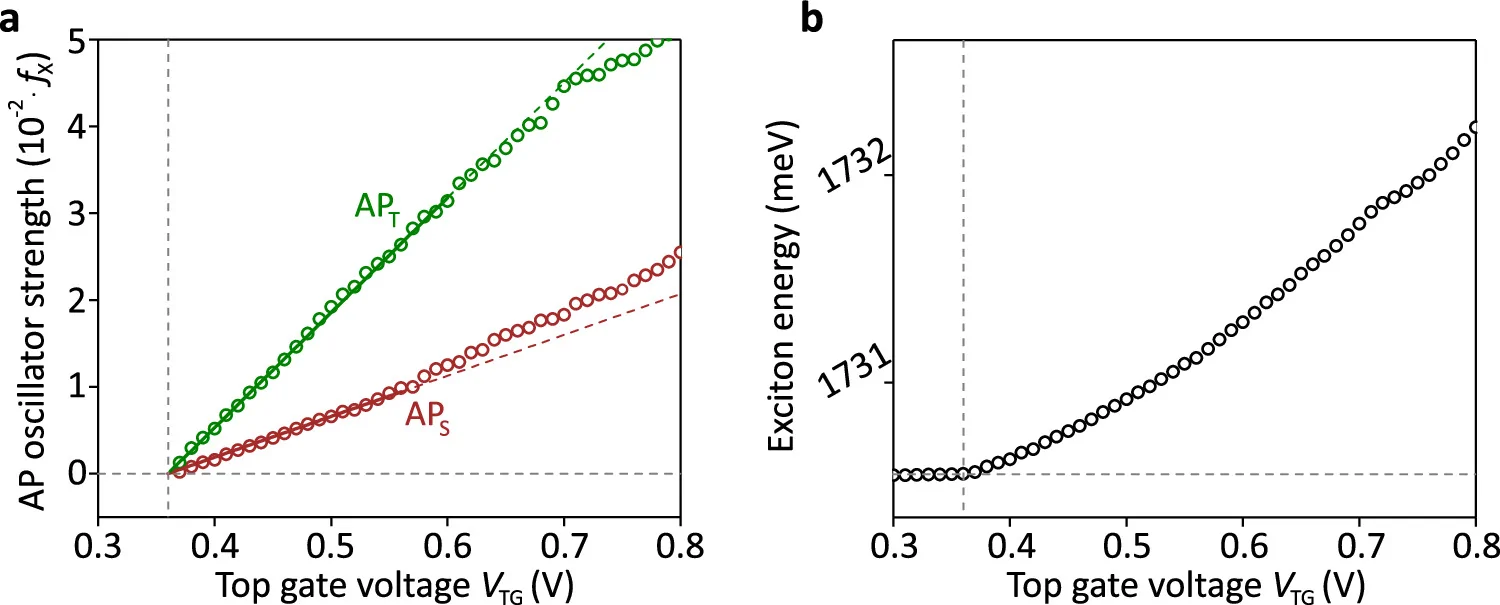
Determination of the doping onset voltage. (**a**) Top-gate voltage dependent spectral weights ${f}_{{{\rm{AP}}}_{{\rm{S}},{\rm{T}}}}$ the AP resonances. The ${f}_{{{\rm{AP}}}_{{\rm{S}},{\rm{T}}}}$ values are determined based on TM fits to the reflectance spectra, and expressed relative to oscillator strength *f*_X_ of the exciton at charge neutrality. The linear fits in the low-density regime (solid lines) are extrapolated to zero spectral weight to obtain *V*_0_. (**b**) The X resonance energy is determined based on dispersive Lorentzian fitting of the X spectral profile as a function of *V*_TG_. The onset of the blueshift, marked by dashed lines, corresponds to *V*_0_.

**Extended Data Fig. 3:**
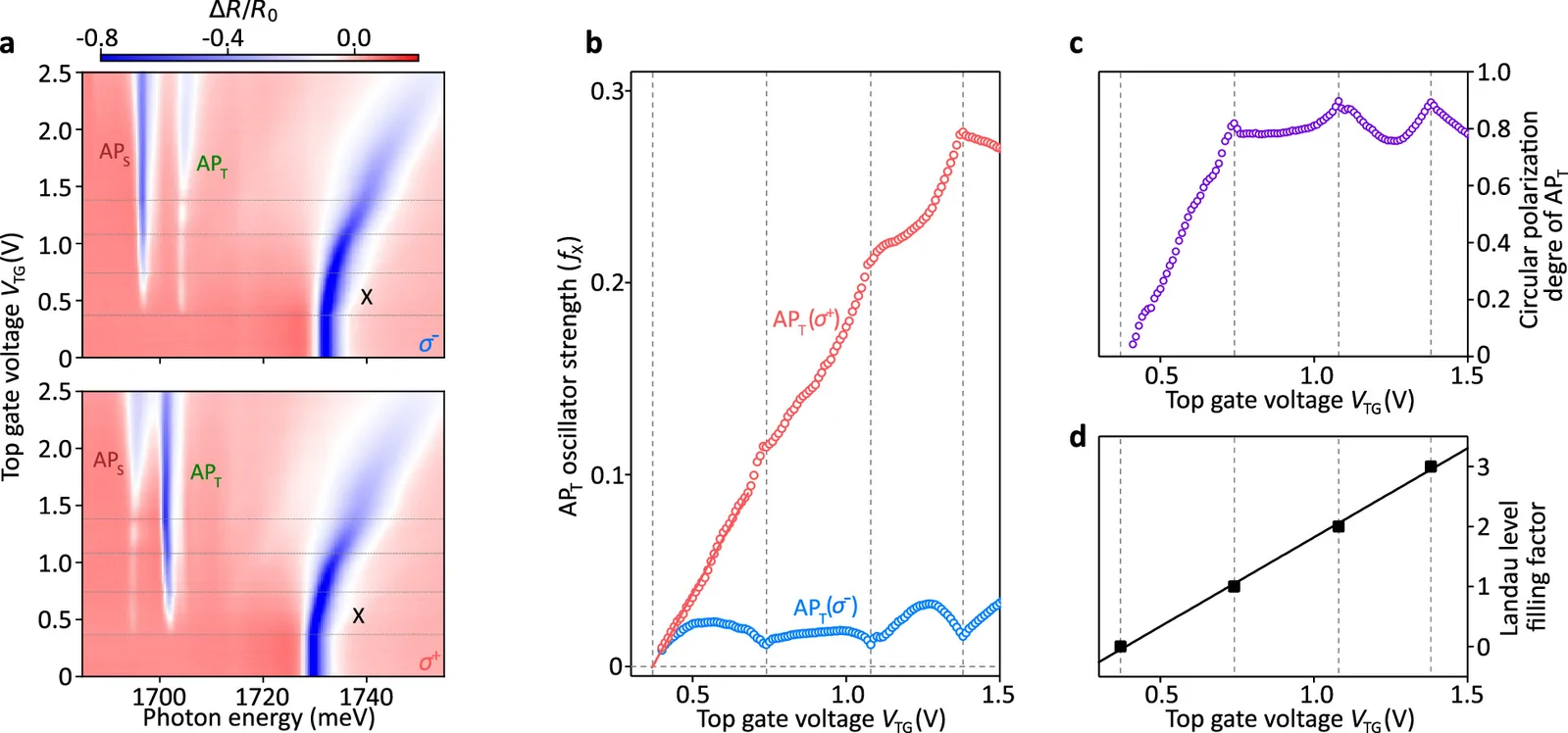
Electron density calibration based on the Shubnikov-de Haas oscillations in the optical spectra. (**a**) *V*_TG_ evolutions of the reflectance contrast spectra acquired in *σ*^−^ (top panel) and *σ*^+^ (bottom panel) polarization at *T* = 1.6 K and *B* = 8 T. (**b**) Gate-voltage dependence of the AP_T_(*σ*^±^) oscillator strength extracted by fitting its spectral profile in *σ*^±^ polarization with the TM model. The value of ${f}_{{{\rm{AP}}}_{{\rm{T}}}}({\sigma }^{\pm })$ is expressed relative to the exciton oscillator strength determined by fitting its spectral profile at charge neutrality (averaged over two circular polarizations). Solid line depicts the linear fit to the voltage-dependent ${f}_{{{\rm{AP}}}_{{\rm{T}}}}({\sigma }^{\pm })$ in the low-density regime, which is used to obtain the *V*_TG_ corresponding to *ν* = 0. (**c**) Circular polarization degree of the AP_T_ resonance determined as ${{\mathcal{P}}}_{{{\rm{AP}}}_{{\rm{T}}}}=[{f}_{{{\rm{AP}}}_{{\rm{T}}}}({\sigma }^{+})-{f}_{{{\rm{AP}}}_{{\rm{T}}}}({\sigma }^{-})]$/$[{f}_{{{\rm{AP}}}_{{\rm{T}}}}({\sigma }^{+})+{f}_{{{\rm{AP}}}_{{\rm{T}}}}({\sigma }^{-})]$. (**d**) *V*_TG_ values corresponding to integer filling factors *ν*. A solid line indicates linear fit with $\nu =({C}_{{\rm{TG}}}/e)\cdot(\frac{h}{eB})\cdot ({V}_{{\rm{TG}}}-{V}_{0})$, whose slope is used to extract the value of *C*_TG_/*e* allowing us to calibrate electron density *n*_*e*_ = (*C*_TG_/*e*) ⋅ (*V*_TG_ − *V*_0_) based on the applied *V*_TG_. Gray dashed lines in all panels mark the voltages at which AP spectral weights exhibit cusps/dips, which correspond to the integer LL filling factors.

Our experiments are performed on two dual-gated WSe_2_ monolayer devices, yielding consistent results (see the ‘Device fabrication’ section). Their optical response is explored in a confocal microscope setup integrated with a magneto-optical cryostat, which enables reaching sample temperatures down to *T* = 1.6 K (see the ‘Experimental setup’ section and Extended Data Fig. 1). In the main text, we focus on the results obtained on the electron-doped side for device A, whose schematic and optical micrograph are depicted in Fig. 1a,b (Supplementary Section 3 provides details for device B). Figure 1c shows a low-temperature resonant reflectance contrast spectrum (Δ*R*/*R*_0_) of our device as a function of *n*_e_, whose value is controlled by applying a top gate voltage *V*_TG_. The dependence of *n*_e_ on *V*_TG_ is precisely calibrated based on the observation of Landau-level (LL) signatures at high magnetic fields^27^ (see Extended Data Figs. 2 and 3 and the ‘Calibration of doping density’ section). Consistent with previous reports^28,29^, we find that upon electron doping, the oscillator strength of the exciton (X) resonance is progressively transferred to two distinct redshifted AP resonances: AP_S_ and AP_T_. They arise from attractive interactions between optically created excitons and the electrons residing in the same and opposite valleys, respectively (Fig. 1d), which are responsible for the formation of singlet (intravalley) and triplet (intervalley) trions^30^. Even though the X resonance in this regime transforms into a repulsive polaron, for simplicity, we refer to it as X, since we focus on the low-density regime. At higher densities (≳ 10^12^ cm^−2^), we observe a third resonance at even lower energies, which is due to the formation of hexcitons (HX)^31^ originating from simultaneous exciton dressing by electrons in both valleys.

**Extended Data Fig. 4:**
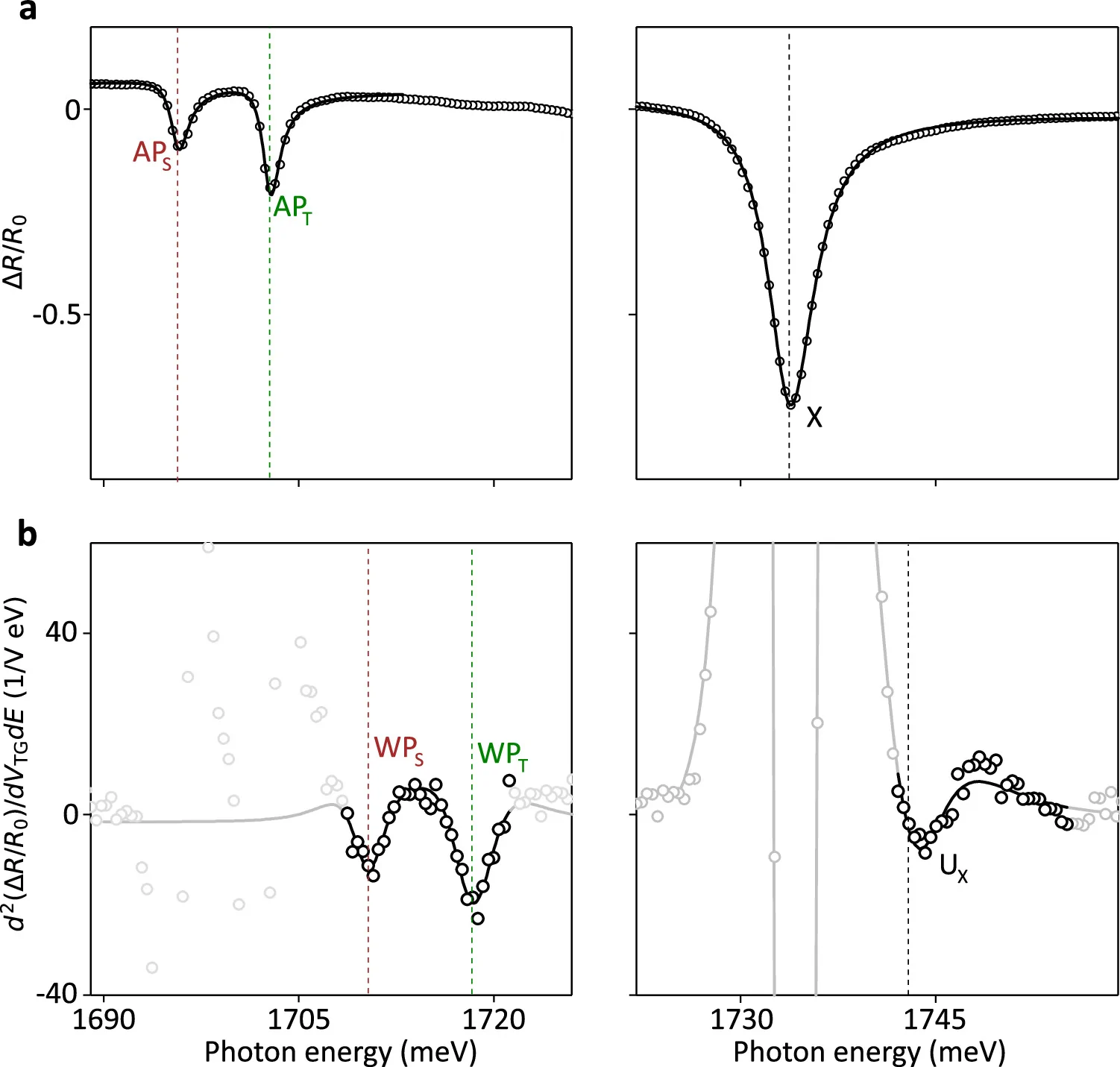
Fitting the spectral profiles of optical transitions. (**a**,**b**) Example reflectance contrast spectra (**a**) and their second derivatives with respect to gate voltage and energy (**b**) plotted in the spectral range of the AP/WP resonances (left) and X/U_X_ resonances (right). The spectra were measured for device A at *T* = 1.6 K, *B* = 0, and *n*_*e*_ ≈ 3.6× 10^11^ cm^−2^. Points mark the experimental data, while solid lines show the fitted lineshapes of optical resonances with the ${\mathcal{L}}$ or ${\mathcal{D}}$ spectral profiles described in ‘Fitting the spectral energies of optical resonances’ section. In the case of umklapp transitions, the black (gray) curves/datapoints lie inside (outside) the spectral window in which the fit was performed. Vertical dashed lines mark the energies of optical transitions extracted from the fitted curves.

Along with these well-established optical resonances, our Δ*R*/*R*_0_ spectra also exhibit a set of weaker features in the low-*n*_e_ regime, where electrons are expected to form a WC^8–14^. These transitions become readily discernible on differentiating the data with respect to both *V*_TG_ and photon energy (*E*), as shown in Fig. 1e (see the ‘Analysis of reflectance spectra’ section). In particular, at high energies, we observe the U_X_ peak that blueshifts relative to X, with the splitting $\Delta {E}_{{{\rm{U}}}_{{\rm{X}}}}$ scaling proportionally to *n*_e_ (Fig. 1f,h; Extended Data Fig. 4 shows the fitting procedures). This is an umklapp signature of the WC^8^ arising due to the Bragg scattering of excitons by the periodic WC potential (Fig. 1g), which leads to an optical excitation at $\Delta {E}_{{{\rm{U}}}_{{\rm{X}}}}\approx {\hslash }^{2}{k}_{{\rm{W}}}^{2}/2{m}_{{\rm{X}}}={h}^{2}{n}_{{\rm{e}}}/\sqrt{3}{m}_{{\rm{X}}}$, where *k*_W_ is the reciprocal lattice constant of the triangular WC and *m*_X_ is the exciton mass. Thus far, such an umklapp resonance has been observed exclusively in MoSe_2_-based systems^8–11,18,24^. Compared with MoSe_2_ monolayers^8^, U_X_ in the present case exhibits an almost twofold larger $\Delta {E}_{{{\rm{U}}}_{{\rm{X}}}}$ splitting at a given *n*_e_. This is due to the lower mass of excitons, which consist of electrons residing in a different spin–orbit-split band than those forming the WC. Specifically, optically active excitonic transitions involve the more dispersive top conduction band, with a theoretically predicted effective mass of ${m}_{{\rm{t}},{\rm{e}}}^{* }\approx 0.3{m}_{0}$ (ref. ^32^), where *m*_0_ is the free electron mass. Together with the hole mass ${m}_{{\rm{h}}}^{* }\approx 0.4{m}_{0}$ (refs. ^32,33^), this yields a relatively low translational exciton mass of ${m}_{{\rm{t}},{\rm{e}}}^{* }+{m}_{{\rm{h}}}^{* }\approx 0.7{m}_{0}$, which remains in excellent agreement with *m*_X_ ≈ 0.68*m*_0_ extracted from our data in Fig. 1h, which is further confirmed by prior electron scattering experiments^34^. By contrast, the WC electrons originate from the less-dispersive bottom conduction band with a larger effective mass (${m}_{{\rm{b}},{\rm{e}}}^{* }\approx 0.4-0.5{m}_{0} > {m}_{{\rm{t}},{\rm{e}}}^{* }$)^32,35^, which nevertheless remains smaller than the mass (~0.7*m*_0_) of WC electrons in MoSe_2_ (refs. ^8,36^). Despite that, the WC in the present system is considerably more stable, persisting up to densities that are roughly twice as high, *n*_c_ ≈ 7 × 10^11^ cm^−2^, as indicated by the disappearance of U_X_. Assuming the above ${m}_{{\rm{b}},{\rm{e}}}^{* }$, the WC melting transition occurs when the ratio of Coulomb to kinetic energies is ${r}_{{\rm{s}}} \sim {m}_{{\rm{b}},{\rm{e}}}^{* }/\sqrt{{n}_{{\rm{e}}}}\approx 10-15$. This is more than two times lower than the theoretical predictions for the clean limit^37^, suggesting an important role of disorder in stabilizing the WC at a reduced *r*_s_ (refs. ^13,38^). This conjecture is further supported by our analysis of spectral broadening of Bragg-scattered polaron resonances (Supplementary Section 1).

In particular, the differentiated spectra in Fig. 1e also display two other weak blueshifting resonances in the energy range between AP and X, which remained elusive in previous studies of the MoSe_2_ platform. The positions of these resonances, WP_S_ and WP_T_, are traced in Fig. 1f. The energy splitting between them is essentially independent of the electron density, and remains close to the exchange-induced splitting between AP_S_ and AP_T_ resonances of around 7 meV. Moreover, both peaks disappear around the same critical density *n*_c_ ≈ 7 × 10^11^ cm^−2^ as the exciton umklapp transition, suggesting that they similarly originate from umklapp scattering of the WC. We first rule out singlet and triplet trions undergoing such an umklapp process. In this scenario, the energy splitting $\Delta {E}_{{{\rm{WP}}}_{{\rm{S}},{\rm{T}}}}$ between a given WP_S,T_ umklapp branch and the corresponding main AP_S,T_ resonance should increase with *n*_e_, with a slope inversely proportional to the translational trion mass (${m}_{{\rm{T}}}={m}_{{\rm{b}},{\rm{e}}}^{* }+{m}_{{\rm{X}}}$). Owing to the aforementioned difference in effective masses between the two spin–orbit-split conduction bands, such an *m*_T_ is expected to be much larger than *m*_X_, suggesting a considerably lower slope of the associated trion umklapp splitting. By contrast, $\Delta {E}_{{{\rm{WP}}}_{{\rm{S}},{\rm{T}}}}$ in our experiments exhibits a slope that is similar to $\Delta {E}_{{{\rm{U}}}_{{\rm{X}}}}$, and can be reproduced assuming the same translational mass of *m*_X_ ≈ 0.68*m*_0_, as shown by the fits in Fig. 1f,h. This observation suggests that WP_S_ and WP_T_ transitions rather arise due to umklapp-scattered neutral excitons that are dressed into WPs by the WC.

**Fig. 2:**
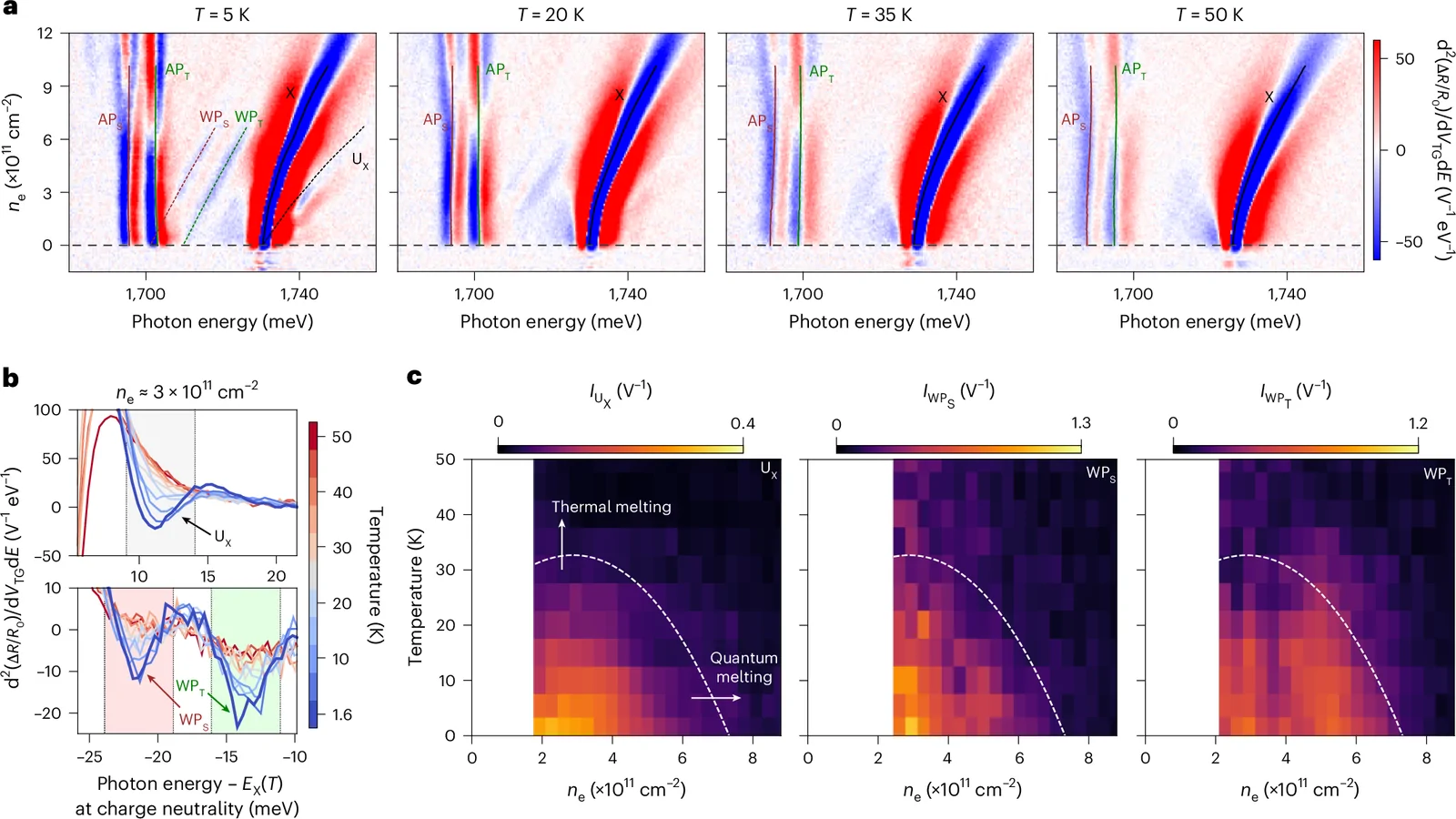
Robustness of the WC to thermal and quantum fluctuations. **a**, *n*_e_ evolutions of differentiated reflectance contrast spectra acquired at various temperatures (as indicated). The solid lines mark the fitted energies of main X, AP_T_ and AP_S_ resonances, whereas the dashed lines represent expected positions of the umklapp peaks at *T* = 5 K. **b**, Linecuts through the plots in **a** (as well as similar datasets from different temperatures), showing the temperature-dependent spectral profiles of the exciton umklapp (top) and WPs (bottom) at *n*_e_ ≈ 3 × 10^11^ cm^−2^. To account for the temperature-induced bandgap shift, the energy axes of all plots are offset by the exciton energy *E*_X_(*T*) at charge neutrality. The shaded areas mark a few-millielectrons-wide energy ranges around the respective umklapp resonances used for determining their spectral weight by averaging background-corrected ∣d^2^(Δ*R*/*R*_0_)/d*V*_TG_d*E*∣ signal (see the ‘Analysis of WC melting’ section). **c**, *T*–*n*_e_ phase diagrams of the WC acquired using U_X_ (left), WP_S_ (middle) and WP_T_ (right). Each map shows the averaged spectral weight of the umklapp resonance divided by the oscillator strength of the corresponding main optical transition. The dome-shaped phase boundary (identical for all plots; dashed line) is a guide to the eye. Source data

**Extended Data Fig. 5:**
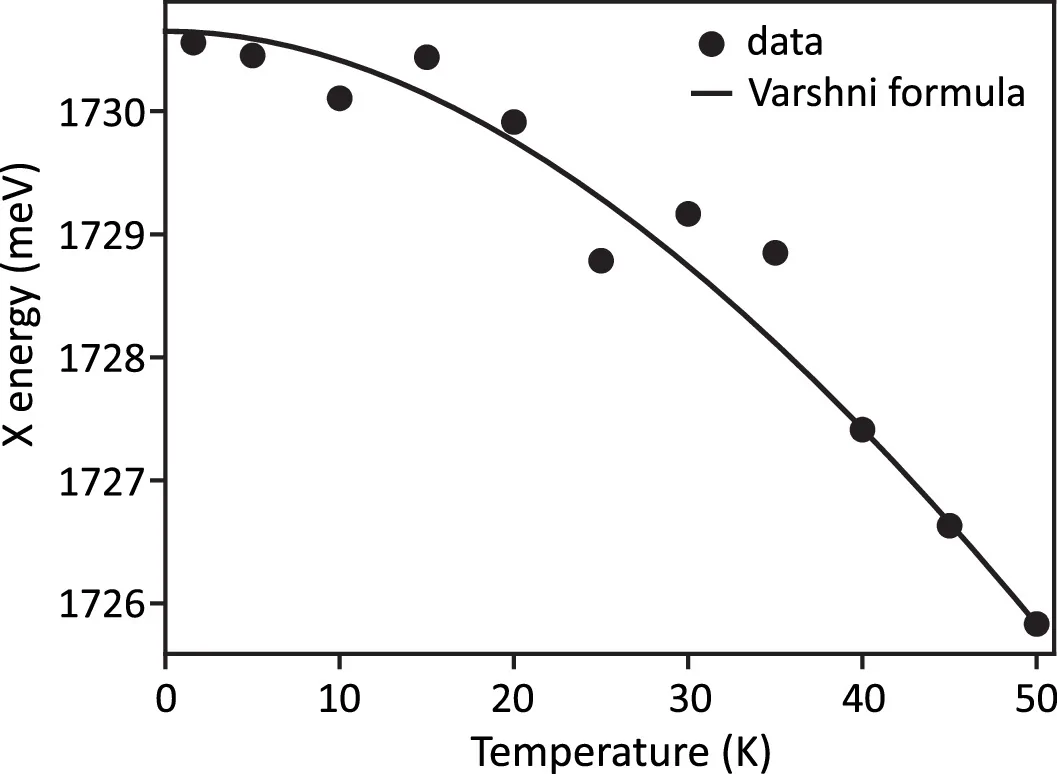
Sample temperature calibration. Extracted X resonance energy at charge neutrality as a function of temperature, overlaid with the curve calculated using the Varshni formula.

In contrast to the previously observed exciton umklapp peak U_X_, the energy splittings $\Delta {E}_{{{\rm{WP}}}_{{\rm{S}},{\rm{T}}}}$ of both WPs do not extrapolate to zero in the low-density limit, but instead exhibit a sizable offset *Δ* (Fig. 1h). Before addressing the origin of this effect, we first provide further evidence for the identification of WPs. To this end, we investigate melting of the WC by tracking *n*_e_ evolutions of differentiated spectra at elevated temperatures (Fig. 2a), where both U_X_ and WP_S,T_ transitions weaken considerably (Extended Data Fig. 5 and the ‘Calibration of the sample temperature’ section provide the temperature calibration details). Since inferring their spectral weights directly from colour-scale maps is hindered by the proximity of strong main exciton transitions, for quantitative analysis, we extract these weights by integrating the background-corrected ∣d^2^(Δ*R*/*R*_0_)/d*V*_TG_d*E*∣ signal in narrow energy windows around each peak (see Fig. 2b and the ‘Analysis of WC melting’ section). The resulting amplitudes are normalized by the oscillator strengths of the corresponding X or AP resonances, and plotted as a function of both *T* and *n*_e_ in Fig. 2c with identical colour scales up to an overall multiplicative factor. Such relative spectral weights are several times larger for WPs, consistent with interaction-induced WP–AP hybridization that does not occur for U_X_. Regardless of this difference, each umklapp branch is observed within similar *T*−*n*_e_ phase space regions, reminiscent of the characteristic dome-shaped phase diagram of the WC^6^. Around the critical density *n*_c_, the electron kinetic energy of a few millielectronvolts greatly exceeds the thermal energy of around 0.1 meV at *T* = 1.6 K, leading to quantum melting of the WC for *n*_e_ ≳ *n*_c_. At higher *T*, thermal fluctuations of the electrons reduce the *n*_e_ range of the WC phase, which ceases to exist above the critical temperature of *T*_m_ ≈ 30 K. This value is about three times larger than that of the WC in a MoSe_2_ monolayer^8–11^, despite similar dielectric screening and a smaller electron effective mass in WSe_2_. This is consistent with our conjecture that the Wigner solid in the present system is stabilized by disorder^39^.

**Fig. 3:**
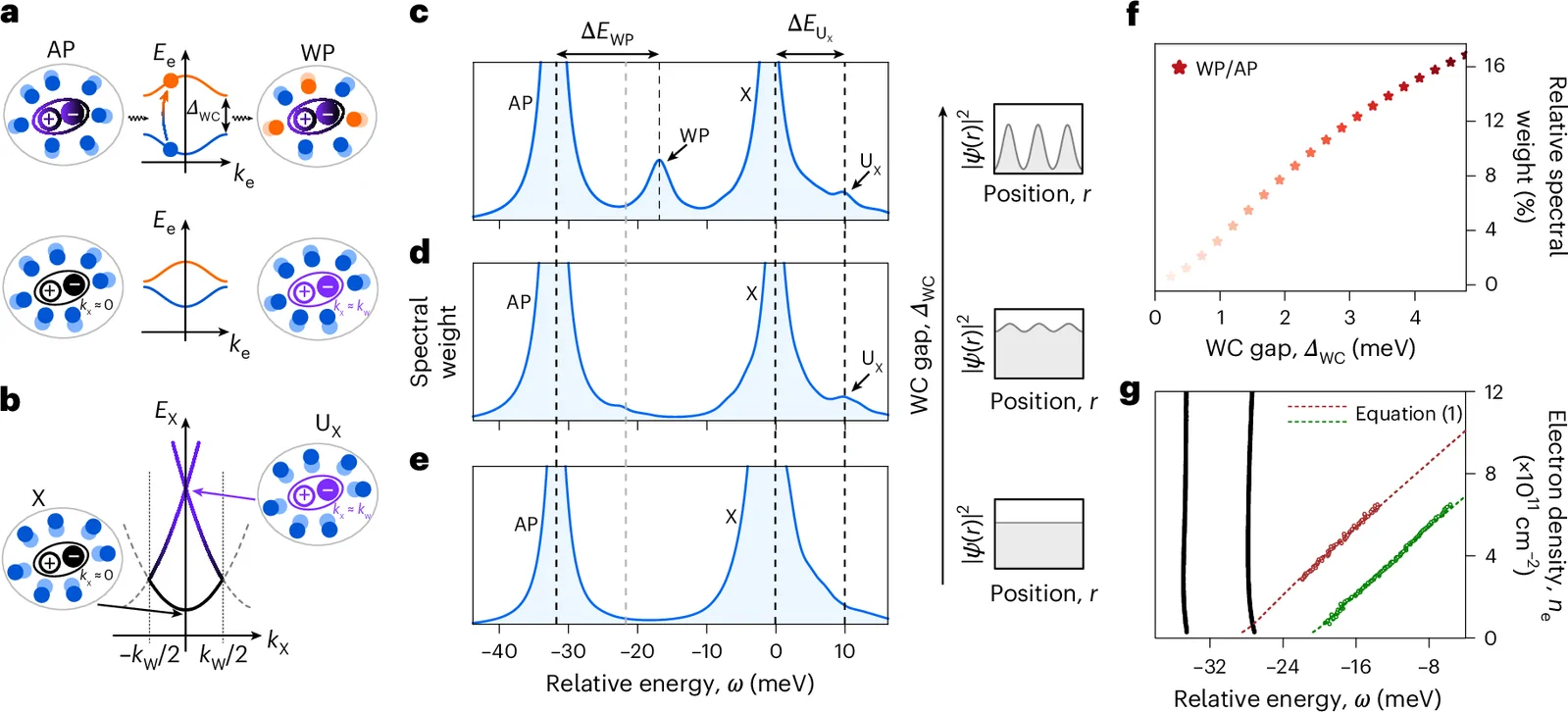
Theoretical description of the WP. **a**, Illustration of AP and WP formation. In the weak-WC limit with a small gap *Δ*_WC_ (bottom), the AP (WP) originates primarily from the particle–hole dressing of a ground (Bragg-scattered) exciton with momentum *k* ≈ 0 (*k* ≈ *k*_W_). For larger *Δ*_WC_ (top), these two excitations hybridize due to strong attractive exciton–electron interactions, mediated by particle–hole excitations across the WC gap. The hybridization results in an enhancement of the WP spectral weight and an increase in its energy detuning from the AP. **b**, Cartoon showing the corresponding exciton umklapp scattering by repulsive exciton–electron interactions, where the energy splitting between the main and umklapp branches is largely independent of *Δ*_WC_. **c**–**e**, Exciton spectral functions calculated using a spin-independent model within the Chevy approximation for a fixed *n*_e_ = 5 × 10^11^ cm^−2^, and various WC gaps: *Δ*_WC_ = 0 (**e**); weak WC with a small gap where U_X_ appears at an energy detuning $\Delta {E}_{{{\rm{U}}}_{{\rm{X}}}}\approx {\hslash }^{2}{k}_{{\rm{W}}}^{2}/2{m}_{{\rm{X}}}$ (**d**); strong WC with a large gap, where WP of a sizable spectral weight appears at an energy splitting $\Delta {E}_{{\rm{WP}}} > \Delta {E}_{{{\rm{U}}}_{{\rm{X}}}}$ (**c**). **f**, Relative spectral weight of the WP compared with the AP calculated as a function of *Δ*_WC_ for a fixed *n*_e_ = 5 × 10^11^ cm^−2^. **g**, *n*_e_-dependent energies of both singlet and triplet AP and WP transitions: points represent the experimental data from Fig. 1, whereas the dashed lines mark the fitted energies of WP_S_ and WP_T_ resonances using a single-parameter effective model given by equation (1). For each WP branch, the energy is obtained by adding the experimentally determined AP energy to the theoretically calculated AP–WP energy splitting at a given *n*_e_. Source data

The above observations unequivocally confirm the connection between electronic crystallization and the emergence of WP resonances in the optical spectra. The mechanism behind their formation is, however, qualitatively distinct from simple Bragg reflection of the excitonic bands that leads to the U_X_ resonance. Specifically, we find that WPs—similar to APs—arise due to strong attractive interactions between the electrons and excitons. As illustrated in Fig. 3a,b, these interactions mediate a hybridization between two polaron branches originating from dressing of the main and Bragg-scattered excitons. The formation of the WP, therefore, involves both exciton Bragg umklapp scattering and coupling to particle–hole excitations across the many-body WC gap *Δ*_WC_ (that should be understood as the mean-field order parameter of the WC). This sets the scale of the effective potential felt by the electrons, and is responsible for the presence of the sizable offset *Δ* between the AP and WP energies in the low-density limit (Fig. 1f,h).

**Extended Data Fig. 6:**
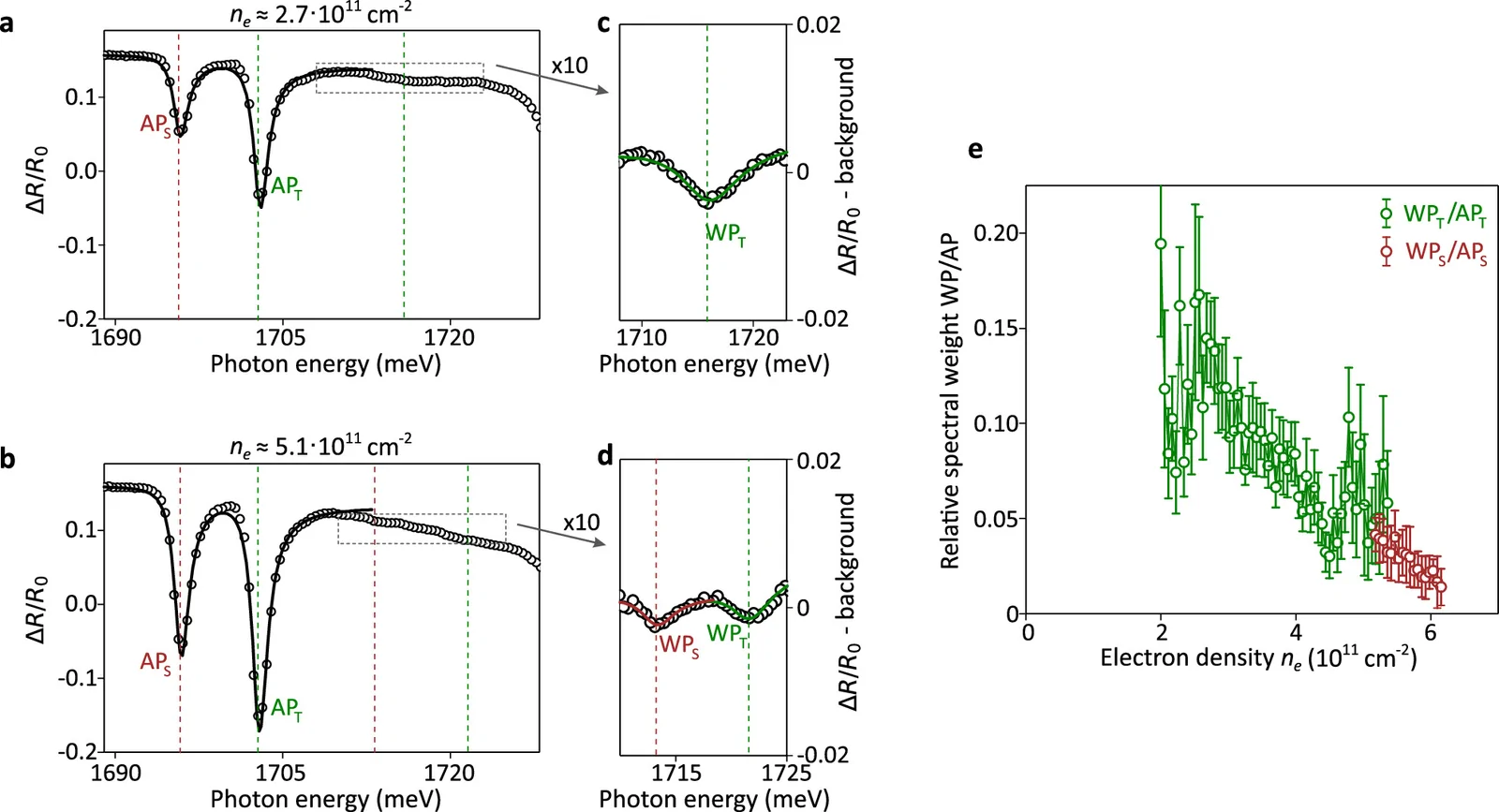
Oscillator strengths of optical resonances based on TM fitting. (**a**,**b**) Example reflectance contrast spectra measured for device A at *T* = 1.6 K, *B* = 0, and two different electron densities (as indicated). The solid lines mark the fits of the AP spectral profiles with the TM model. (**c**,**d**) Close-ups of the WP energy ranges (marked by dashed rectangles in panels (**a**,**b**)), showing background-corrected spectra together with the corresponding TM fits of the WP spectral profiles. (**e**) Relative oscillator strengths of WP_T_ and WP_S_ resonances divided by the spectral weights of corresponding main AP resonances as a function of electron density (error bars correspond to standard errors of the fit). Note that determination of WP intensities is possible only when they are spectrally well-separated from X/AP resonances.

To obtain an effective understanding of the resulting optical spectra, we build on refs. ^40,41^, and describe polaron dressing within the Chevy approximation^42^. We assume spin-independent density–density interactions between electrons and excitons (Supplementary Sections 4 and 7). The WC is treated on a mean-field level as a spontaneously stabilized charge-density wave, with *Δ*_WC_ serving as a tuning parameter. As expected, for *Δ*_WC_ = 0 (that is, in the absence of a WC), our model yields two resonances—X and AP (Fig. 3e). For small *Δ*_WC_ > 0, an exciton umklapp U_X_ appears in the calculated spectra (Fig. 3d) at an energy detuning $\Delta {E}_{{{\rm{U}}}_{{\rm{X}}}}$ largely determined by the reciprocal WC lattice constant: this is a weak-WC regime explored in previous studies of MoSe_2_ monolayers, where only U_X_, but no WP, is observed^8–11^. By contrast, we find that the WP becomes discernible only for larger *Δ*_WC_ (Fig. 3c). Moreover, although $\Delta {E}_{{{\rm{U}}}_{{\rm{X}}}}$ remains largely insensitive to *Δ*_WC_ in this regime (for a given *n*_e_), both AP–WP splitting and relative spectral weight of the WP increase substantially with *Δ*_WC_ (Fig. 3c,f). Inferring *Δ*_WC_ from the WC melting temperature of *k*_B_*T*_m_ ≈ 3 meV, the calculated splitting between the AP and WP at *n*_e_ ≈ 5 × 10^11^ cm^−2^ is roughly 15 meV (Fig. 3b), which is in good agreement with the measurement (Fig. 1h). These predictions not only explain the larger energy detuning of the WP resonances but also their sizable oscillator strengths, which we estimate to be 10%−20% of the APs based on spectra measured at low *T* and *n*_e_ (see the ‘Determination of spectral weights of optical resonances’ section and Extended Data Fig. 6).

These theoretical observations can be captured by a simplified analytical model describing interaction-mediated hybridization between AP and WP resonances:

1 $${H}_{{\rm{hyb}}}=\left(\begin{array}{ll}{E}_{{\rm{AP}}}&{V}_{{\rm{hyb}}}({n}_{{\rm{e}}})\\ {V}_{\,\mathrm{hyb}\,}^{\,* }({n}_{{\rm{e}}})&{E}_{{\rm{AP}}}+\frac{{h}^{2}}{\sqrt{3}}\frac{{n}_{{\rm{e}}}}{{m}_{{\rm{X}}}}\end{array}\right),$$

where the diagonal terms represent the energies of ‘bare’ AP and WP states that correspond to *k* ≈ 0 and *k* ≈ *k*_W_ excitons dressed into polarons; *V*_hyb_(*n*_e_) denotes hybridization between these states, which scales linearly with the exciton–electron interaction strength and increases for larger *Δ*_WC_. Since the overall AP energy *E*_AP_ is difficult to predict theoretically, we focus on the splitting between AP and WP. As shown in Fig. 3g, the minimal model given by equation (1) reproduces the measured *n*_e_ dependence of the splittings $\Delta {E}_{{{\rm{WP}}}_{{\rm{S,T}}}}$ using a single free parameter (Supplementary Section 5). In the experimentally inaccessible low-density regime (*n*_e_ ≲ 10^11^ cm^−2^), the predicted splitting is primarily determined by the hybridization gap ∣*V*_hyb_(*n*_e_)∣, which decreases rapidly as *n*_e_ is lowered. At higher densities, the diagonal Bragg-scattering term proportional to *n*_e_/*m*_X_ dominates, leading to a linear increase in the splitting with *n*_e_. In this regime, the hybridization between AP_S,T_ and WP_S,T_ depends only weakly on *n*_e_, manifesting as an effective energy offset *Δ* in phenomenological fits from Fig. 1h (Supplementary Section 4 discusses a complementary theoretical treatment proposed in refs. ^43,44^).

**Fig. 4:**
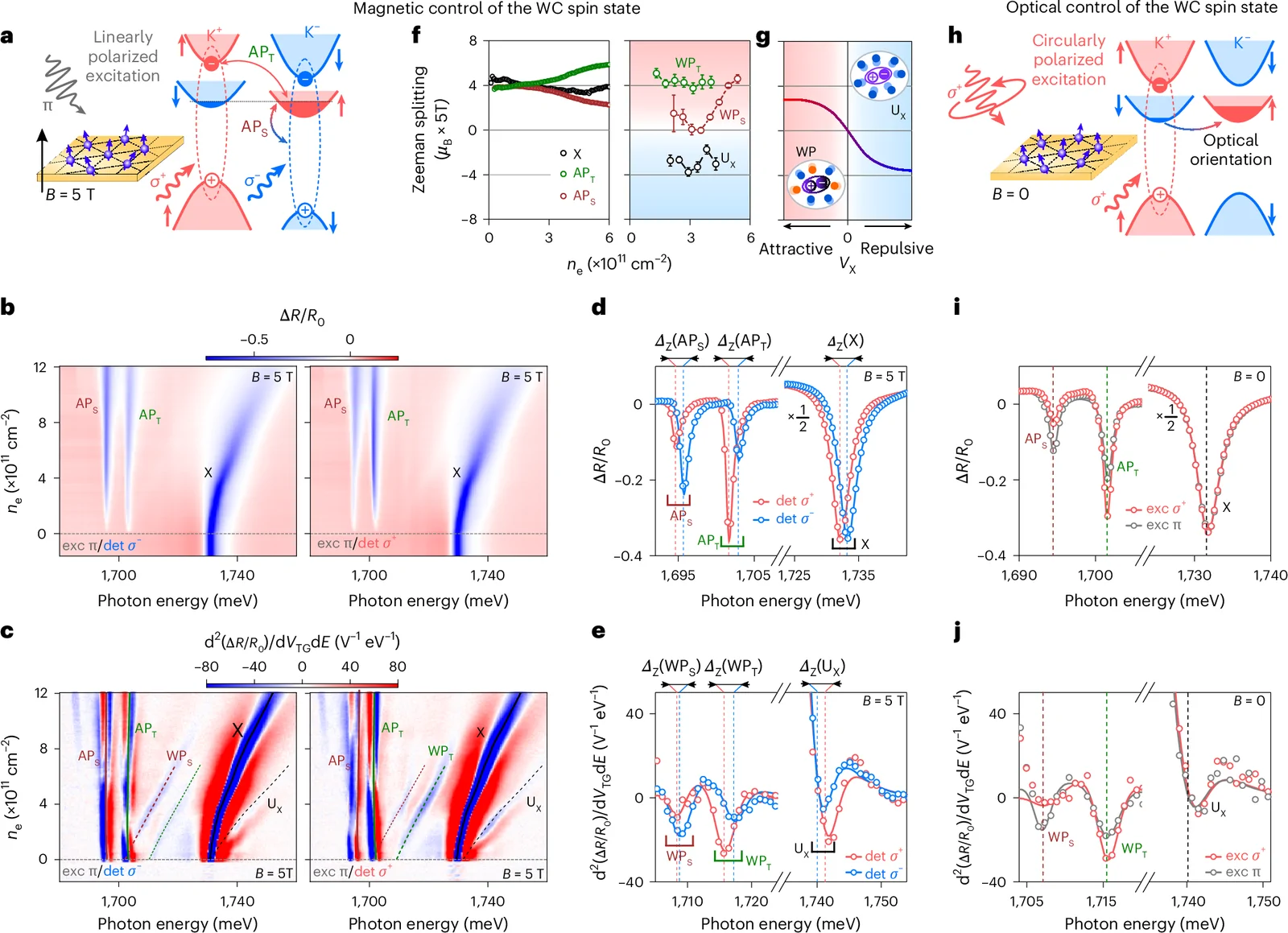
Magnetic and optical control of the WC spin state. **a**,**h**, Schematics illustrating two approaches for inducing sizable spin-valley polarization of the WC: by applying a magnetic field (**a**) or by illuminating the sample with circularly polarized light at *B* = 0 (**h**). **b**,**c**, *n*_e_ evolutions of reflectance contrast (**b**) and its second derivative (**c**). The spectra were measured under linearly polarized excitation in two circular polarizations of detection at *B* = 5 T and *T* = 1.6 K. The solid lines show the fitted energies of the main optical transitions, whereas the dashed lines mark the expected positions of the umklapp resonances determined by fitting their splitting from the corresponding co-polarized main exciton transitions with ${h}^{2}{n}_{{\rm{e}}}/\sqrt{3}{m}_{{\rm{X}}}+\Delta$ for a fixed *m*_X_ = 0.68*m*_0_ and different offsets *Δ*. **d**,**e**, Linecuts through the maps in **b** and **c** at *n*_e_ ≈ 3 × 10^11^ cm^−2^ showing polarization-resolved main resonances (**d**) and the corresponding umklapp transitions (**e**). The data in **e** were binned along the energy axis. The solid lines show the fitted spectral profiles, whereas the dashed ones mark the extracted peak energies (see the ‘Fitting the spectral energies of optical resonances’ section for details about the fitting procedures). **f**, Zeeman splittings between *σ*^−^- and *σ*^+^-polarized branches of the main excitons (left) and the corresponding umklapp resonances (right) determined as a function of *n*_e_ at *B* = 5 T (the latter data were binned in Δ*n*_e_ ≈ 0.5 × 10^11^ cm^−2^ increments and their error bars represent standard deviations). **g**, Calculated Zeeman splitting of the umklapp-scattered exciton as a function of exciton–electron interaction strength *V*_X_ (Supplementary Section 6). The splitting is positive (negative) for attractive (repulsive) interactions, consistent with the experimental results. **i**,**j**, Similar spectra as in **d** (**i**) and **e** (**j**) at the same *n*_e_, but acquired at *B* = 0 using two different polarizations of excitation: *σ*^+^ and linear (with co-polarized detection in both cases). The difference in intensities of the triplet and singlet branches of AP/WP transitions demonstrates optical spin pumping of the WC (Supplementary Section 2). Source data

Having elaborated on the nature of WP transitions, we now explore their spectral response when the WC becomes spin-valley polarized. To induce such polarization, we pursue two different approaches. In the first one, we apply an external magnetic field, which lifts the degeneracy of the two conduction bands (Fig. 4a) due to the valley Zeeman effect^45,46^. Figure 4b,c shows the circular-polarization-resolved evolutions of bare and differentiated reflectance spectra at *B* = 5 T. Owing to the intra- and intervalley dressing of AP_S_ and AP_T_ resonances, the large field-induced electronic spin orientation manifests as a sizable circular polarization degree of APs, with AP_S_ (AP_T_) stronger in *σ*^−^ (*σ*^+^) helicity, as clearly shown in the linecuts in Fig. 4d. We find that WP_S_ and WP_T_ resonances exhibit the same behaviour as the respective APs (Fig. 4e), further confirming that they arise from the polaron dressing of excitons by electrons in the same and opposite valleys, respectively.

In the second approach, the spins of WC electrons are optically oriented with circularly polarized light (Fig. 4h), giving rise to a similar change in the WP intensities even at *B* = 0 (Fig. 4i,j). As discussed in Supplementary Section 2, this optical spin-pumping method^47^ enables sizable spin-polarization degrees of the WC without affecting its charge-order parameter, thereby paving the way for future dynamical spin-control experiments.

**Extended Data Fig. 7:**
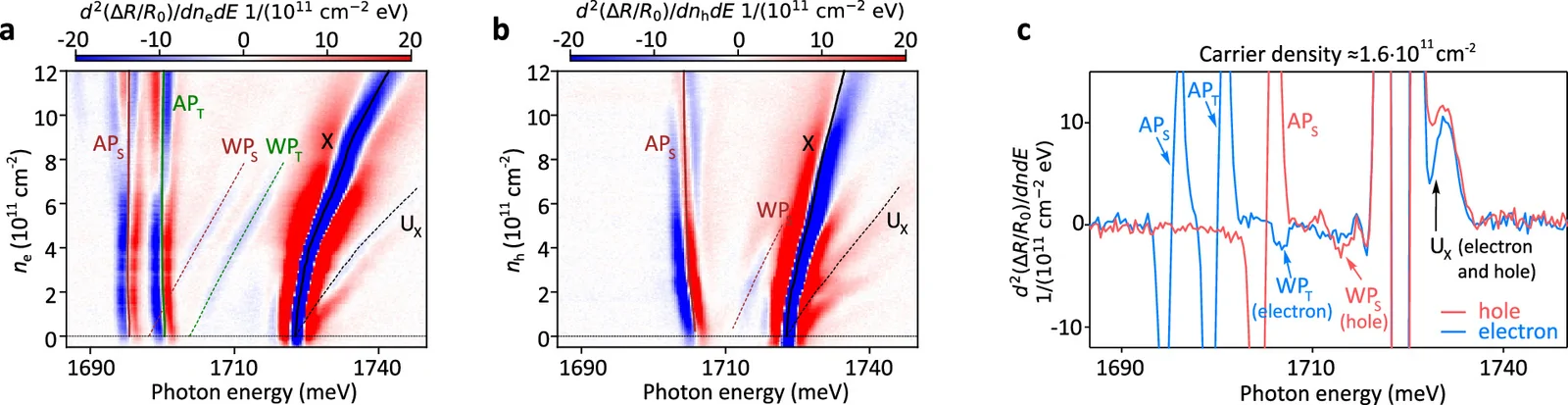
Comparison of optical responses of electron and hole WCs. (**a**,**b**) Differentiated reflectance contrast spectra measured for device A at *T* = 1.6 K and *B* = 0 as a function of electron (**a**) and hole (**b**) densities. The solid lines mark the fitted energies of AP and X resonances, while dashed lines represent the expected positions of the U_X_ and WP_S,T_ transitions calculated assuming that their energy splitting from the corresponding main resonance is given by $\Delta +{h}^{2}{n}_{{\rm{e}},{\rm{h}}}/\sqrt{3}{m}_{{\rm{X}}}$ with the same *m*_X_ = 0.68*m*_0_ and *Δ* = 0 (*Δ* > 0) for U_X_ (WP) resonances. (**c**) Linecuts through the maps in panels (**a**,**b**) showing two differentiated spectra at electron and hole densities of about 1.6 × 10^11^ cm^−2^.

Independent of whether the WC is magnetically or optically oriented, the energy detunings $\Delta {E}_{{{\rm{WP}}}_{{\rm{S}},{\rm{T}}}}$ of both WP branches from the corresponding AP transitions exhibit very similar doping dependence to that in the spin-unpolarized case. This observation indicates that the singlet–triplet fine structure of exciton–polarons does not play an important role in the WP formation. This is further supported by our zero-field spectroscopy of hole WCs in the same device (see the ‘Comparison between optical signatures of electron and hole WCs’ section and Extended Data Fig. 7), where only a singlet trion is bound, resulting in the presence of a single pair of AP_S_ and WP_S_ branches. Although the spectral proximity of the X resonance precludes the unequivocal identification of hole WP in these measurements, the results suggest that WPs are universal light–matter excitations of sufficiently robust WCs, irrespective of the trion spin structure.

**Extended Data Fig. 8:**
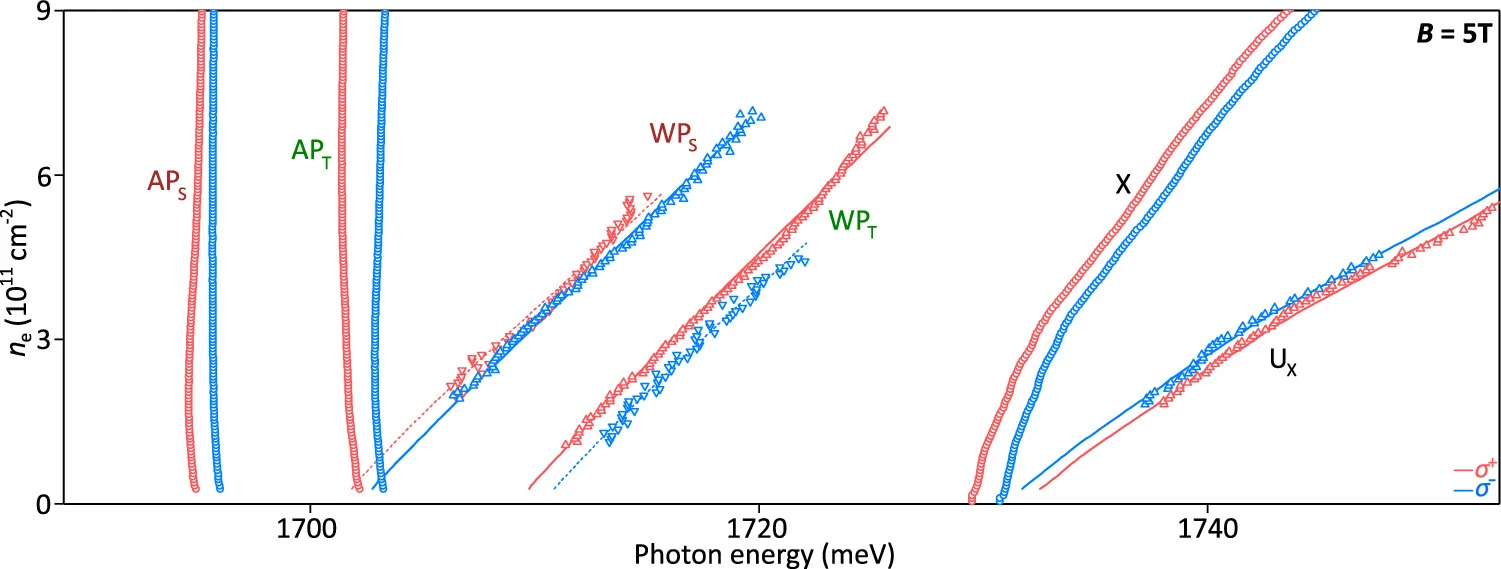
Energies of main and umklapp resonances at *B* = 5 T. Electron-density-dependent energies of both main and umklapp transitions determined at *B* = 5 T in the two circular polarizations by fitting lineshapes of these resonances in bare or differentiated reflectance contrast spectra from Fig. 4b,c. Solid lines mark the expected umklapp energies determined by fitting their splittings from the corresponding co-polarized main excitonic transitions as *h*^2^*n*_e,h_/$\sqrt{3}{m}_{{\rm{X}}}+\Delta$ with fixed exciton mass *m*_X_ = 0.68*m*_0_ and various offsets *Δ*.

Finally, we explore the Zeeman splitting of WP resonances on the electron-doped side at *B* = 5 T. As shown in Fig. 4d–f and Extended Data Fig. 8, these splittings for both WPs have the same sign as those of the X and AP transitions. This contrasts sharply with U_X_, for which the energy order of *σ*^+^- and *σ*^−^-polarized branches—and hence the sign of the Zeeman splitting—is reversed (Fig. 4e,f). For the main excitonic transitions, the splitting is governed predominantly by the Zeeman effect of the bright neutral exciton, yielding *Δ*_Z_ ≈ 4*μ*_B_*B* (where *μ*_B_ denotes the Bohr magneton)^45,46^. Conversely, the umklapp transitions are expected to exhibit smaller *Δ*_Z_ due to momentum-dependent electron–hole exchange interaction, which suppresses the Zeeman effect for finite-momentum Bragg-umklapp-scattered excitons^8,18^. Although our spin-dependent calculations (Supplementary Section 6) confirm these expectations, they also reveal that exciton–electron interactions *V*_X_ can induce a Zeeman splitting for the umklapp peaks, whose magnitude increases with ∣*V*_X_∣, and approaches that of the bare exciton for sufficiently strong interactions (Fig. 4g). The sign of this interaction-induced splitting is directly determined by the character of the interactions: it is the same (opposite) as that of the X Zeeman splitting for attractive (repulsive) *V*_X_. This is consistent with our experimental observations, confirming that WPs result from the attractive particle–hole dressing of Bragg-scattered excitons by the WC, further highlighting the central role of exciton–electron interactions in the investigated system. We note that unlike WP_T_, the Zeeman splitting *Δ*_Z_(WP_S_) of the singlet WP branch exhibits a complex, non-monotonic dependence on electron density. This behaviour is probably linked to the spin-valley polarization degree of the electron system, which shows a local maximum at which *Δ*_Z_(WP_S_) reaches a minimum, suggesting that WP transitions could serve as sensitive probes of emergent spin phenomena in the electron crystal.

Our experiments demonstrate that optical excitations in WCs can not only sense electronic order but also strongly interact with it, becoming dressed into WPs. The spectral properties of these quasiparticles are governed by collective excitations of the WC, rendering them powerful probes of the WC dynamics. This sensitivity may allow the optical monitoring of the WC gap, which could be tuned at a fixed electron density by a proximal metallic screening layer^48,49^, even at ultrafast timescales^50^.

More broadly, the framework established in our work provides a route to exploring hybrid light–matter excitations in a wide range of strongly correlated electronic phases—including fractional Chern insulators^51,52^, kinetically driven magnetic orders^53^ and superconductors^54,55^—opening possibilities for optically accessing the fine structure of collective modes in strongly interacting quantum matter.

## Methods

### Device fabrication

The flakes used for device fabrication were mechanically exfoliated from bulk crystals (HQ Graphene, WSe_2_, NIMS hexagonal boron nitride (hBN), NGS natural graphite) onto silicon substrates. They were stacked together inside a N_2_-filled glovebox using a standard dry-transfer method^56^. In this technique, the flakes are aligned with submicrometre precision and subsequently picked up with a transparent polymer stamp consisting of a dome-shaped polydimethylsiloxane (PDMS) covered with a thin polycarbonate (PC) film. The finished vdW heterostructure was then released onto a silicon substrate with a 285-nm-thick SiO_2_ layer and prepatterned electrodes (approximately 15-nm gold with about 5-nm titanium adhesion layer). Before the release step, the device was aligned with respect to the electrode pattern such that each few-layer graphene flake (top gate, contact and back gate) was connected to a separate electrode. Finally, the PC residue was removed by dissolving it in either chloroform or dichloromethane.

For both devices, the thicknesses of the encapsulating hBN layers were selected to ensure either constructive (device A) or destructive (device B) interference between resonant light reflected from various interfaces away from the WSe_2_ monolayer region. This choice guarantees almost Lorentzian lineshapes of excitonic optical transitions in the WSe_2_ monolayer reflectance contrast spectra, which greatly facilitates resolving weak spectral signatures of the WC. In particular, device A, along with two approximately 30-nm-thick hBN layers separating WSe_2_ from the top and bottom few-layer graphene gates, consisted of a third ~40-nm-thick hBN capping layer (Fig. 1a). By contrast, no hBN capping layer was used for device B. For both samples, the doping density in the WSe_2_ monolayer was tuned by applying top-gate voltage *V*_TG_, keeping the bottom gate at the ground potential. Device A showed no signatures of electrical hysteresis when *V*_TG_ was ramped in a loop. To avoid residual gate hysteresis for device B (Supplementary Figs. 3 and 4), its charge configuration was reset before each measurement by sweeping the gate voltage to –4 V with the imaging light on.

### Experimental setup

Our experiments were carried out in a closed-cycle cryostat equipped with a superconducting magnet (up to 9 T). The sample was mounted on *x*–*y*–*z* piezoelectric stages inside a variable-temperature insert (VTI) filled with He exchange gas to ensure efficient thermal contact, allowing the sample temperature to be tuned from 1.6 K to room temperature. Reflectance measurements were performed in a confocal geometry (Extended Data Fig. 1) using a supercontinuum laser (30-MHz repetition rate) coupled to a motorized filter and a single-mode fibre, enabling the automatic selection of excitation spectral range. The excitation light was collimated before entering the VTI through a wedged window, and then focused onto the sample surface onto a diffraction-limited spot by a microscope objective with a large numerical aperture (0.8). The excitation power in front of the VTI window was monitored with a fast photodiode and actively stabilized using a proportional–integral–derivative (PID) controller coupled to a voltage-controlled variable optical attenuator. Except for the optical spin orientation experiments, the power value was kept in the range of a few tens of nanowatts to ensure that the excitons did not perturb the probed electronic system. The light reflected from the sample was collected by the same objective, directed into the detection path by a beamsplitter, coupled into a single-mode fibre, and finally dispersed by a spectrometer equipped with a Peltier-cooled charge-coupled device (CCD) camera, enabling recording of the spectra. The polarizations of both excitation and detection beams were controlled with a set of linear polarizers and achromatic waveplates.

### Analysis of reflectance spectra

To determine Δ*R*/*R*_0_, the measured reflectance spectrum *R* is normalized using the reference spectrum *R*_0_ acquired in a region of the device featuring all layers except for the WSe_2_ monolayer. The reflectance contrast is then determined at each photon energy as *R*_c_ ≡ Δ*R*/*R*_0_ = ((*R* − *C*_dark_) − (*R*_0_ − *C*_dark_))/(*R*_0_ − *C*_dark_), where *C*_dark_ denotes the dark counts of the CCD camera.

The derivative of the reflectance contrast with respect to the energy and gate voltage was obtained using a standard symmetric difference quotient method. To reduce the noise, the numerical derivative is approximated as $R{\prime} ({V}_{{\rm{G}},n})=\left[\left({R}_{{\rm{c}}}\right.({V}_{{\rm{G}},n+\delta })-{R}_{{\rm{c}}}({V}_{{\rm{G}},n-\delta })\right]/\left[{V}_{{\rm{G}},n+\delta }-{V}_{{\rm{G}},n-\delta }\right]$ and $R{\prime} ({E}_{n})=\left[\left({R}_{{\rm{c}}}\right.({E}_{n+\delta {\prime} })-{R}_{{\rm{c}}}({E}_{n-\delta {\prime} })\right]/\left[{E}_{n+\delta {\prime} }-{E}_{n-\delta {\prime} }\right]$, where the index *n* denotes subsequent points^8^. The step *δ* in gate voltage and $\delta {\prime}$ in energy are chosen such that noise from abrupt changes in *R*_c_ occurring within Δ*n*_e_ of a few 10^10^ cm^−2^ or Δ*E* ≲ 1 meV is suppressed.

### Fitting the spectral energies of optical resonances

To determine the energies of the main excitonic transitions (X, AP_S_ and AP_T_) in the reflectance contrast spectra, we fit their spectral profiles with the following dispersive Lorentzian formula^8,27,57^

2 $$\begin{array}{l}{\mathcal{L}}({E}_{0},A,\gamma ,\varphi ;E\,)=A\cos \varphi \frac{\gamma /2}{{(E-{E}_{0})}^{2}+{\gamma }^{2}/4}+A\sin \varphi \frac{{E}_{0}-E}{{(E-{E}_{0})}^{2}+{\gamma }^{2}/4}+B(E\,),\end{array}$$

where *E* denotes the photon energy, whereas *A*, *E*_0_ and *γ* represent the amplitude, peak energy and linewidth of the analysed optical resonance, respectively. The parameter *φ* represents the phase shift induced by the interference of light reflected off different interfaces within the heterostructure, which depends on both resonance energy and amplitude. Finally, *B*(*E*) accounts for a background term stemming from spectral proximity of other resonances, which is assumed to be either constant *B*(*E*) = *C* or polynomially dependent on the photon energy. For the APs, we assume that both transitions have a common phase. Moreover, at densities below ~10^11^ cm^−2^—where the APs become very weak—their phase and linewidths are fixed at values obtained for a higher *n*_e_. Coherent with previous works^8,27,57^, this method allows us to precisely reproduce the lineshapes of all three main resonances (Extended Data Fig. 4a).

Owing to their markedly smaller intensities, the umklapp-scattered exciton and WP resonances cannot be fitted using the above procedure. Instead, to extract their spectral positions, we directly fit the differentiated d^2^(Δ*R*/*R*_0_)/d*V*_TG_d*E* spectra, where the umklapp transitions are greatly amplified due to their much larger blueshifts compared with the main excitonic resonances. To quantitatively describe the resulting spectral profiles, we assume that the evolution of the U_X_ or WP lineshape ${{\mathcal{L}}}_{{\rm{U}}}({E}_{{\rm{U}}},{A}_{{\rm{U}}},{\gamma }_{{\rm{U}}},{\varphi }_{{\rm{U}}};E)$ is dominated by the linear-in-density energy blueshift. This allows us to approximate its differentiated spectral profile ${{\mathcal{D}}}_{{\rm{U}}}({E}_{{\rm{U}}},{A}_{{\rm{U}}},{\gamma }_{{\rm{U}}},{\varphi }_{{\rm{U}}};E)$ in the following way:

3 $$\begin{array}{l}{{\mathcal{D}}}_{{\rm{U}}}({E}_{{\rm{U}}},{A}_{{\rm{U}}},{\gamma }_{{\rm{U}}},{\varphi }_{{\rm{U}}};E\,)\\=\displaystyle\frac{{d}^{\,2}}{d{V}_{{\rm{TG}}}dE}{{\mathcal{L}}}_{{\rm{U}}}({E}_{{\rm{U}}},{A}_{{\rm{U}}},{\gamma }_{{\rm{U}}},{\varphi }_{{\rm{U}}};E\,)\\\,\approx \left(\displaystyle\frac{d{E}_{{\rm{U}}}}{d{{\rm{V}}}_{{\rm{TG}}}}\right)\times \displaystyle\frac{\partial }{\partial {E}_{{\rm{U}}}}\displaystyle\frac{d}{dE}{{\mathcal{L}}}_{{\rm{U}}}({E}_{{\rm{U}}},{A}_{{\rm{U}}},{\gamma }_{{\rm{U}}},{\varphi }_{{\rm{U}}};E\,)\\ \,={\tilde{A}}_{{\rm{U}}}\displaystyle\frac{\cos {\varphi }_{{\rm{U}}}\left[{\gamma }_{{\rm{U}}}^{2}/4-3{(E-{E}_{{\rm{U}}})}^{2}\right]{\gamma }_{{\rm{U}}}/2+\sin {\varphi }_{{\rm{U}}}(E-{E}_{{\rm{U}}})\left[-3{\gamma }_{{\rm{U}}}^{2}/4+{(E-{E}_{{\rm{U}}})}^{2}\right]}{{\left[{\gamma }_{{\rm{U}}}^{2}/4+{(E-{E}_{{\rm{U}}})}^{2}\right]}^{3}},\end{array}$$

where ${\tilde{A}}_{{\rm{U}}}=2{A}_{{\rm{U}}}\times d{E}_{{\rm{U}}}/d{V}_{{\rm{TG}}}$ is the effective amplitude of the analysed umklapp transition in the differentiated spectrum, whereas *γ*_U_, *φ*_U_ and *E*_U_ denote its linewidth, phase and energy, respectively. In view of their negligible amplitudes, the phases of all three umklapp resonances can be fixed at the same value corresponding to the low-oscillator-strength limit of the phase of an optical resonance in the analysed device^8^. It is determined based on the transfer-matrix (TM) simulations of the device reflectivity spectrum^58,59^ (described in the next section) and further confirmed by the phase of two AP resonances in the low-density limit, yielding *φ*_U_ ≈ 3.0 for device A and *φ*_U_ ≈ 0.3 for device B. To facilitate the fitting procedure, the linewidths of the umklapp resonances are always constrained within reasonable ranges, and assumed to increase with *n*_e_ for U_X_. The fitting itself is carried out within a narrow spectral window around a given resonance. In the case of WPs, each transition is first individually fitted. The obtained parameters are then partially constrained and used as a starting point for a final fit of the two WP branches with the sum of two ${{\mathcal{D}}}_{{\rm{U}}}$ profiles that is further corrected by a background term accounting for the tails of the main X and AP resonances in the spectrum. This is justified by their relatively large energy distance from both WP resonances. This assumption does not hold for the U_X_ resonance, which spectrally overlaps with the main exciton resonance. To account for this, the U_X_ is fitted using a sum of its expected profile ${{\mathcal{D}}}_{{\rm{U}}}$ and numerically computed second derivative of the dispersive Lorentzian profile fitted beforehand to the X resonance. As shown in Extended Data Fig. 4b, these procedures enable us to accurately reproduce the spectra of both WP and U_X_ transitions and quantitatively extract their energies.

### Determination of spectral weights of optical resonances

Although dispersive Lorentzian fitting described in the earlier section allows for a precise determination of the spectral positions of optical resonances, the amplitudes obtained in such a way do not directly correspond to the oscillator strengths. Whenever we are interested in the latter (for normalizing the WC phase diagrams (Fig. 2c and Supplementary Fig. 4c) and for analysing the density-dependent AP intensities (Extended Data Figs. 2 and 3)), we use a more complex TM model^58–61^. In this case, each resonance is described with the following complex susceptibility: *χ*(*E*) = −(*ℏc*/*E*_0_)*γ*_rad_/(*E* − *E*_0_ + i*γ*_nrad_/2), where *E* is the photon energy; *E*_0_ is the resonance energy; and *γ*_nrad_/*ℏ* and *γ*_rad_/*ℏ* denote its non-radiative and free-space radiative decay rates, respectively, with the latter being directly proportional to the oscillator strength. Assuming the hBN thicknesses from the ‘Device fabrication’ section and standard values of hBN/graphene/SiO_2_ refractive indices^8,61^, we can aptly reproduce the interference pattern within the vdW heterostructure, thereby correctly capturing the lineshapes of optical resonances, as shown for the example spectra in Extended Data Fig. 6a,b. In these fits, analogously to the case of dispersive Lorentzian profile fitting, the TM-calculated spectra are overlaid on a background term. Moreover, non-radiative broadening *γ*_nrad_ controlling the linewidths of APs is assumed to be the same for both singlet and triplet branches, and fixed in the low-density regime below a few 10^10^ cm^−2^. By performing such fitting at various electron densities, we can directly determine *n*_e_-dependent oscillator strengths of the main AP/X resonances as *f*_AP/X_(*n*_e_) = *γ*_rad,AP/X_(*n*_e_)/*γ*_rad,X_ relative to that of the X at charge neutrality (set by *γ*_rad,X_).

Given their inherently weak nature, WP transitions cannot be easily fitted with a similar TM model, particularly near the WC melting point. In this regime, tracing WP intensities requires alternative approaches (see the ‘Analysis of WC melting’ section) that do not allow for quantitative extraction of *f*_WP_. This is, however, possible at low temperatures in a selected density range in which the WC is stable and WP transitions are spectrally well separated from the X/AP resonances. To facilitate the TM fitting in this regime, we first subtract the fitted AP and X spectral profiles, as well as similarly corrected reflectance spectrum measured at charge neutrality (to suppress the influence of residual etaloning in our setup). These data are then further corrected by subtracting a linearly dependent background fitted in the spectral range between X and AP_T_. Finally, the WP resonances in the resulting background-corrected spectrum are fitted using the above-described TM model with fixed energies obtained based on the dispersive Lorentzian model. As shown in Extended Data Fig. 6c,d, this procedure reproduces the WP lineshapes reasonably well, allowing us to extract their density-dependent spectral weights *f*_WP_/*f*_AP_ relative to the corresponding AP branches (Extended Data Fig. 6e). Despite the fact that their peak-to-peak amplitudes are more than a few tens of times smaller than that of APs, the WPs exhibit sizably larger linewidth, presumably due to additional broadening channels stemming from the WC disorder (Supplementary Section 1). For this reason, *f*_WP_/*f*_AP_ can be as high as 10%–20% in the low-density regime, supporting our conclusion of the substantial hybridization of AP and WP transitions. Still, we stress that in view of complex background subtraction procedures, the extracted *f*_WP_/*f*_AP_ values are fraught with large uncertainties, which are probably larger than the statistical error bars shown in Extended Data Fig. 6e.

### Calibration of doping density

Since the energy splitting between the U_X_/WP transitions and the corresponding main X/AP resonances is determined by the WC lattice constant, which, in turn, depends on *n*_e_, it is crucial for our analysis to accurately determine *n*_e_. To this end, we describe our device as a parallel-plate capacitor, which allows us to express *n*_e_ as *n*_e_ = (*C*_TG_/*e*) × (*V*_TG_ − *V*_0_), where *e* is an elementary charge, *V*_0_ is the voltage corresponding to the onset of electron filling into the conduction band and *C*_TG_ is the geometrical capacitance between the top gate and WSe_2_ monolayer per unit area of the device.

To determine *V*_0_, we analyse the *V*_TG_-dependent AP oscillator strengths and X energies that are extracted based on TM or dispersive Lorentzian fitting of their spectral profiles (see the ‘Fitting the spectral energies of optical resonances’ and ‘Determination of spectral weights of optical resonances’ sections). Consistent with the Fermi polaron model^17,62^, the spectral weights of singlet and triplet AP resonances exhibit a linear increase with *V*_TG_ in the low-density regime (Extended Data Fig. 2a shows example results obtained at *T* = 1.6 K). Importantly, linear fits to both increasing trends extrapolate to the zero oscillator strength at exactly the same voltage, which precisely corresponds to *V*_0_. This value is consistent with that corresponding to the onset of doping-induced X blueshift (Extended Data Fig. 2b).

The capacitance *C*_TG_ is, in turn, extracted based on Shubnikov–de Haas oscillations of the optical transitions at a high magnetic field (8 T). These oscillations arise from the successive filling of LLs (Extended Data Fig. 3a), giving rise to characteristic cusps in energies and intensities of exciton–polaron transitions at integer LL filling factors^27,61,63^. In our analysis, we focus on the low-density regime in which the electrons primarily fill the states in a single valley, and trace the local maxima (minima) of AP_T_ oscillator strength in *σ*^+^ (*σ*^−^) polarization (Extended Data Fig. 3b,c). Together with *V*_TG_ corresponding to the onset of electron doping, this allows us to determine gate voltages corresponding to subsequent *ν* = 0, 1, 2, 3. As shown in Extended Data Fig. 3d, they are very well reproduced by a linear relation *ν* = (*C*_TG_/*e*) × (*h*/*eB*) × (*V*_TG_ − *V*_0_), enabling us to extract *C*_TG_/*e* = (5.9 ± 0.3) × 10^11^ cm^−2^ V^−1^. Importantly, this result agrees well with the geometrical capacitance calculated based on the hBN thickness of 30 nm and the hBN dielectric constant of *ϵ*_hBN_ = 3.1 (refs. ^64,65^).

### Calibration of the sample temperature

For all measurements, the sample temperature was determined using the temperature sensor of the VTI. To verify this calibration, we analyse the temperature-induced redshift in the exciton resonance, which arises from the shrinkage of the semiconducting bandgap *E*_g_(*T*) and is well described by the Varshni formula^66^:

4 $${E}_{{\rm{g}}}(T\,)={E}_{0}-\frac{\alpha {T}^{2}}{T+\beta },$$

where *E*_0_ denotes the low-temperature limit, and *α* and *β* are material-specific parameters previously reported as *α* = 0.424 meV K^−1^ and *β* = 170 K for monolayer WSe_2_ (ref. ^67^). As shown in Extended Data Fig. 5, this dependence accurately reproduces the temperature evolution of the exciton energy at charge neutrality in our device, with *E*_0_ serving as the only fitting parameter. The excellent agreement confirms the reliability of our temperature calibration.

### Analysis of WC melting

To determine the temperature- and doping-dependent intensities of umklapp and WP resonances, we pursue a similar approach to that introduced in our previous study^8^. In the first step, U_X_/WP spectral weights at a given *T* and *n*_e_ are estimated by integrating out the absolute value of background-corrected differentiated reflectance signal ∣d^2^(Δ*R*/*R*_0_)/d*V*_TG_d*E* − *B*(*E*, *n*_e_)∣ over a narrow window ${E}_{\min } < E < {E}_{\max }$ around each resonance. The background *B*(*E*, *n*_e_) is obtained separately for U_X_/WP at each *n*_e_ by averaging polynomial profiles fitted to the data at high temperatures (above 35 K), where the umklapp features become indiscernible. To facilitate a quantitative comparison between different U_X_/WP branches, the integration windows are taken to be equally wide for each of them. Moreover, their spectral positions are adjusted to compensate for doping- and temperature-induced shifts of umklapp transitions. In particular, at a given *n*_e_, the windows are displaced according to the temperature-induced energy shift of the neutral exciton *E*_X_(*T*) at charge neutrality (Fig. 2b and Supplementary Fig. 4b).

In the second step, the above-determined spectral weights of umklapp resonances are normalized with the fitted oscillator strengths *f*(*n*_e_, *T*) of the corresponding main X/AP resonances. This finally allows us to calculate the relative intensities of exciton umklapp or WP transitions as

5 $${I}_{{\rm{U}}}({n}_{{\rm{e}}},T\,)=\frac{\int_{{E}_{\min }}^{{E}_{\max }}\left\vert\frac{{d}^{\,2}(\Delta R/{R}_{0})}{d{V}_{{\rm{TG}}}\,dE}-B(E,{n}_{{\rm{e}}})\right\vert{dE}}{f({n}_{{\rm{e}}},T\,)}.$$

The extracted values of *I*_U_(*n*_e_, *T*) for U_X_ and both WP_S,T_ are plotted as a function of *n*_e_ and *T* in Fig. 2c and Supplementary Fig. 4c for devices A and B, respectively, revealing the WC phase diagrams.

### Comparison between optical signatures of electron and hole WCs

To gain further insights into the mechanisms of the Wigner polaron formation, we perform measurements under hole doping in device A. Extended Data Fig. 7a,b shows a representative comparison of the low-temperature reflectance spectra measured as a function of electron (*n*_e_) and hole (*n*_h_) densities (both calibrated using the procedure described in the ‘Calibration of doping density’ section). In both cases, the spectra are plotted using the same colour scale and differentiated with respect to the energy and respective carrier density (rather than the gate voltage), ensuring a consistent correspondence between colour and differentiated signal.

The key difference between the electron and hole optical responses is the presence of only one AP_S_ resonance in the hole spectra. This stems from the opposite ordering of spin-orbit-split bands in the valence band (Fig. 1d), which allows only a singlet hole trion to be bound. By contrast, the low-density evolution of the X resonance is similar for both carrier species, in each case displaying the familiar Bragg umklapp signature U_X_ of WC formation. In general, the hole U_X_ exhibits similar detuning from the main exciton determined by the same *m*_X_ ≈ 0.68*m*_0_ (see also the linecut in Extended Data Fig. 7c), yet showing a non-trivial fine structure at *n*_h_ ≳ 3 × 10^11^ cm^−2^ before completely disappearing around a critical density comparable with that of the electron WC. Although understanding the origin of this fine structure requires further studies, the fact the hole WC persists to much larger densities than ~1 × 10^11^ cm^−2^ reported in ref. ^12^ for ultraclean flux-grown WSe_2_ monolayers further supports our conjecture on the role of disorder in stabilizing both electron and hole crystalline phases in our devices.

Alongside the U_X_ transition, also for hole doping, the spectra exhibit a faint blueshifting resonance (WP_S_) in the energy range between AP_S_ and X, whose properties align with the Wigner polaron picture. In particular, it appears only at low densities. Moreover, its energy splitting from the AP_S_ exhibits a linear increase ${\varDelta }_{{\rm{h}}}+{h}^{2}{n}_{{\rm{h}}}/\sqrt{3}{m}_{{\rm{X}}}$ (Extended Data Fig. 7b, dashed line) with the slope determined by the exciton mass *m*_X_ and a similar zero-density energy offset *Δ*_h_ ≈ 7 meV as for the WPs on the electron side. These observations are consistent with our interpretation that WPs are not limited to a specific spin structure of optical excitations in electron-doped WSe_2_, but rather are general excitations of sufficiently robust WCs. We stress, however, that the unequivocal identification of hole WP_S_ as a WP is hindered by the relatively small binding energy of the hole AP_S_, which causes the WP_S_ transition to appear in the tail of X already at very low hole densities. The spectral overlap between these two resonances increases for *n*_h_ ≳ 3 × 10^11^ cm^−2^, rendering WP_S_ no longer discernible in this regime. Consequently, direct evidence for the WP formation for a setting in which only one trion is bound merits further studies of other transition metal dichalcogenide monolayers.

## Online content

Any methods, additional references, Nature Portfolio reporting summaries, source data, extended data, supplementary information, acknowledgements, peer review information; details of author contributions and competing interests; and statements of data and code availability are available at https://doi.org/10.1038/s41567-026-03395-0.

## Supplementary information

Supplementary Information Supplementary Sections 1–7 and Figs. 1–5 contain additional experimental results (analysis of the role of disorder, efficiency of optical spin orientation and data obtained on device B) and a detailed description of the developed theory model.

## Source data

Source Data Fig. 1 Source data for Fig. 1.

Source Data Fig. 2 Source data for Fig. 2.

Source Data Fig. 3 Source data for Fig. 3.

Source Data Fig. 4 Source data for Fig. 4.

## Data Availability

The data that support the findings of this study are available via Zenodo at https://doi.org/10.5281/zenodo.20273182 (ref. ^68^). Source data are provided with this paper.
