# Supplementary material for: Spectroscopy of Wigner crystal polarons in an atomically thin semiconductor
Source: Nat Phys. 2026 Aug 11;22(11):1776–82. doi: 10.1038/s41567-026-03395-0 (PMC13759297; doi:10.1038/s41567-026-03395-0)
Supplement: Supplementary file 1 [file 41567_2026_3395_MOESM1_ESM.pdf]

nature physics

Supplementary information

https://doi.org/10.1038/s41567-026-03395-0

Spectroscopy of Wigner crystal polarons in an atomically thin semiconductor

In the format provided by the authors and unedited

Supplementary Information for
"Spectroscopy of Wigner crystal polarons in an atomically thin semiconductor"

CONTENTS

S1. Role of disorder in the WC formation 2

S2. Optical spin orientation of the Wigner crystal 3

S3. Data for the device B 4

S4. Theory: Polaron formation 5

S5. Theory: Hybridization model 7

S6. Theory: Magnetic field dependence 8

S7. Theory: Discussion of singlet and triplet AP 9

References 10

S1. ROLE OF DISORDER IN THE WC FORMATION

As discussed in the main text, the WC in the investigated WSe_2 monolayers is exceptionally stable, persisting to large electron densities that correspond to r_s as low as $10-15$. Based on numerous studies of electronic solids in other platforms [S1–S3], including recent scanning tunneling microscopy (STM) experiments on TMD-based systems [S4, S5], we attribute this WC enhancement to the effects of disorder, which can support nucleation of local crystalline order. Such disorder pinning suppresses long-range order, markedly elevating the critical density threshold for the formation of a WC with short-range correlations [S6, S7]. A direct consequence of this process is an increase in WC melting temperature, consistent with the high $T_{\rm m} \approx 30$ K revealed by our experiments. Concurrently, the finite WC correlation length $\xi_{\rm corr}$ introduces an uncertainty in the reciprocal lattice vector $k_{\rm W}$ within the optical excitation spot, which gives rise to a broadening of excitonic umklapp resonances, as shown in Ref. [S8] and schematically illustrated in Fig. S1**a**.

We find that similar disorder-driven broadening also affects the WP resonances. Fig. S1**b** compares theoretically calculated spectral functions of AP and WP resonances for WCs with various $\xi_{\rm corr}$ at $n_{\rm e} \approx 5 \cdot 10^{11}$ cm^{-2}. To facilitate comparison with experiment, the resonances in all calculated spectra are subject to a fixed broadening, which sets the AP linewidth in the clean WC limit to approximately 1.5 meV. The effects of disorder are approximated by assuming that the WC lattice constant follows a Gaussian distribution with mean $a_{\rm WC}$ and standard deviation $\sigma_{\rm d}$, corresponding to a correlation length $\xi_{\rm corr} \sim a_{\rm WC}^2/2\sigma_{\rm d}$. The spectral function of a disordered WC is then obtained by averaging over disorder realizations, corresponding to different lattice constants drawn from this distribution. As expected, while the AP linewidth is barely affected by this WC disorder, the WP broadens substantially with decreasing $\xi_{\rm corr}$. We note that already in the clean limit ($\xi_{\rm corr} = \infty$), the WP is about two times broader than the AP due to many-body effects, but its linewidth further increases by a few times for a disordered WC with $\xi_{\rm corr}$ of a few WC lattice constants. Although our simplified model neglects possible interference effects between WC regions with different lattice constants, these results demonstrate that WP signatures persist even for WCs with suppressed long-range order.

Consistent with these theoretical predictions, the WP resonances in our experiments exhibit markedly larger linewidth compared to their AP counterparts. To analyze this effect, we employ the TM formalism discussed in Methods Sec. 5 to extract nonradiative broadening $\gamma_{\rm nrad}^{\rm WP}$ of the triplet WP, taking advantage of its prominent separation from other resonances in the optical spectra. As seen in Fig. S1**c**, this energy is a few times larger compared to $\gamma_{\rm nrad}^{\rm AP}(n_{\rm e})$ obtained for the corresponding $\rm AP_T$ resonance. Assuming that excessive WP broadening $\Delta\gamma_{\rm nrad} = \gamma_{\rm nrad}^{\rm WP} - \gamma_{\rm nrad}^{\rm AP}$ arises exclusively due to the WC disorder, it can be related to a local variation $\delta k_{\rm W}$ of the reciprocal WC lattice vector as $\Delta\gamma_{\rm nrad} \sim \delta k_{\rm W} \cdot \partial(\Delta E_{\rm WP_T}/\partial k_{\rm W})$. Given that $\Delta E_{\rm WP_T} \approx \Delta_{\rm T} + \Delta E_{\rm U_X}$ where $\Delta E_{\rm U_X} = \hbar^2 k_{\rm W}^2/2m_{\rm X}$ denotes kinetic energy of Bragg-scattered exciton, we obtain $\delta k_{\rm W}/k_{\rm W} \sim \Delta\gamma_{\rm nrad}/2\Delta E_{\rm U_X}$. In view of the existence of additional WP broadening channels, such as those arising from many-body interactions, the inverse of this quantity provides only an approximate lower bound for the WC correlation length $\xi_{\rm corr}/a_{\rm WC}$. As shown in Fig. S1**d**, this value is of the order unity at low densities and increases for larger $n_{\rm e}$, where the WC unit cell shrinks and disorder effects become less prominent. While these observations are qualitatively consistent with our disorder-averaged Chevy calculations, we stress that the obtained values likely provide only rough estimates of the actual WC correlation length scales,

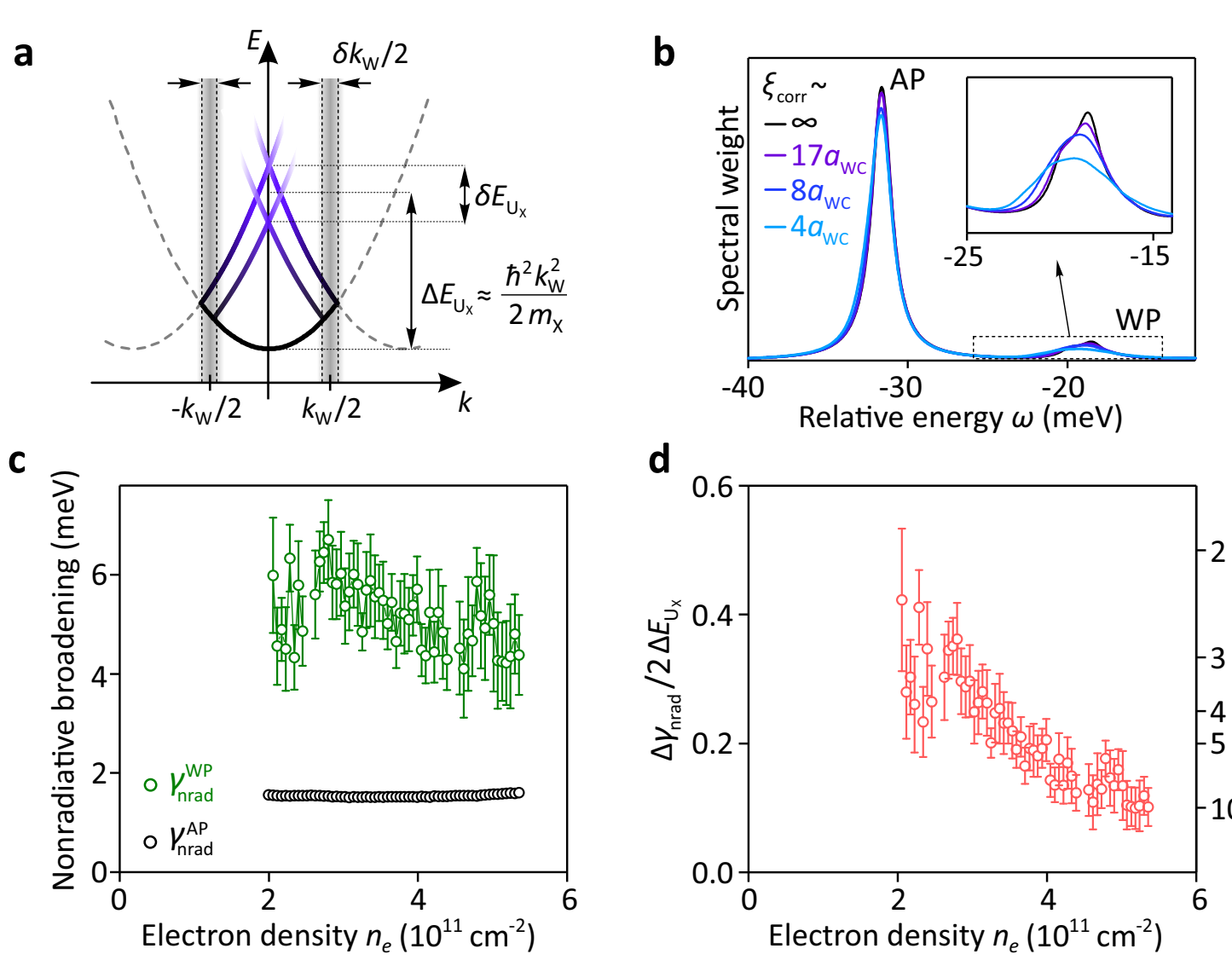

Figure S1. **Influence of WC disorder on the WP broadening.** (**a**) Cartoon illustrating the mechanism of disorder-induced broadening of Bragg-umklapp WC signatures: a finite distribution of the WC reciprocal lattice vectors $\delta k_{\rm W}$ results in a spread of Bragg-umklapp scattering energies. (**b**) Theoretically calculated spectral functions of WP and AP transitions for a WC with Gaussian disorder and different correlation lengths $\xi_{\rm corr}$. To facilitate the comparison with experiment, in these calculations all resonances are subject to additional, disorder-independent broadening determining the AP linewidth in the clean WC limit. (**c**) Density-dependent nonradiative broadenings of the triplet branches of AP and WP resonances obtained by fitting their lineshapes in the spectra from Fig. 1**e** with the TM model. (**d**) Ratio of the difference between WP and AP nonradiative broadenings to twice the exciton Umklapp energy at $k = k_{\rm W}$.

given the number of assumptions and simplifications in our model. Nevertheless, this analysis already supports the influence of disorder on the WC in the investigated WSe_2 system.

S2. OPTICAL SPIN ORIENTATION OF THE WIGNER CRYSTAL

As described in the main text, the spins of the electrons forming a WC can be optically oriented even in the absence of a magnetic field. This is achieved by illuminating the sample with circularly polarized light (Figs. 4**h-j** in the main text and Fig. S2**a**), following an approach similar to that used in previous studies of charge-tunable WSe_2 monolayers [S9, S10]. In contrast to these earlier works, which relied on non-resonant excitation, here we employ a broadband white light resonant with the main optical transitions in the WSe_2 monolayer spectrum. Fig. S2**b** shows density-dependent reflectance contrast spectra measured with such light of linear and circular polarization (using co-polarized detection). While in the former case the two $AP_{T,S}$ resonances exhibit similar intensities, circular excitation drastically enhances their intensity difference in the low electron-density regime. Specifically, the AP_T resonance becomes considerably stronger, whereas the AP_S resonance is markedly weaker compared to the linearly excited spectra, as clearly seen in the linecut in Fig. S2**d**. This observation provides direct evidence of optical spin orientation of the electrons, which are pumped out of the optically driven valley under circularly polarized excitation [S9]. Crucially, we find that this optical spin orientation does not compromise the long-range charge order of the WC, as directly revealed by the presence of exciton-umklapp resonances in the circularly-excited differentiated spectra (Fig. S2**c**). Importantly, while the U_X transitions exhibit the same intensities in both linearly and circularly excited spectra (Figs. S2**e**), the singlet and triplet branches of the WPs undergo the same changes as their AP counterparts, as highlighted in the main text.

Fig. S2**f** shows the spin–valley polarization degree $\langle S_z \rangle$ of the optically oriented electron system as a function of electron density for different excitation powers. As expected for optical spin pumping, $\langle S_z \rangle$ at a given n_e typically increases with increasing excitation power. In parallel, higher $\langle S_z \rangle$ values are achieved at lower n_e, presumably due to longer spin–valley relaxation times of the electrons in such a regime [S10].

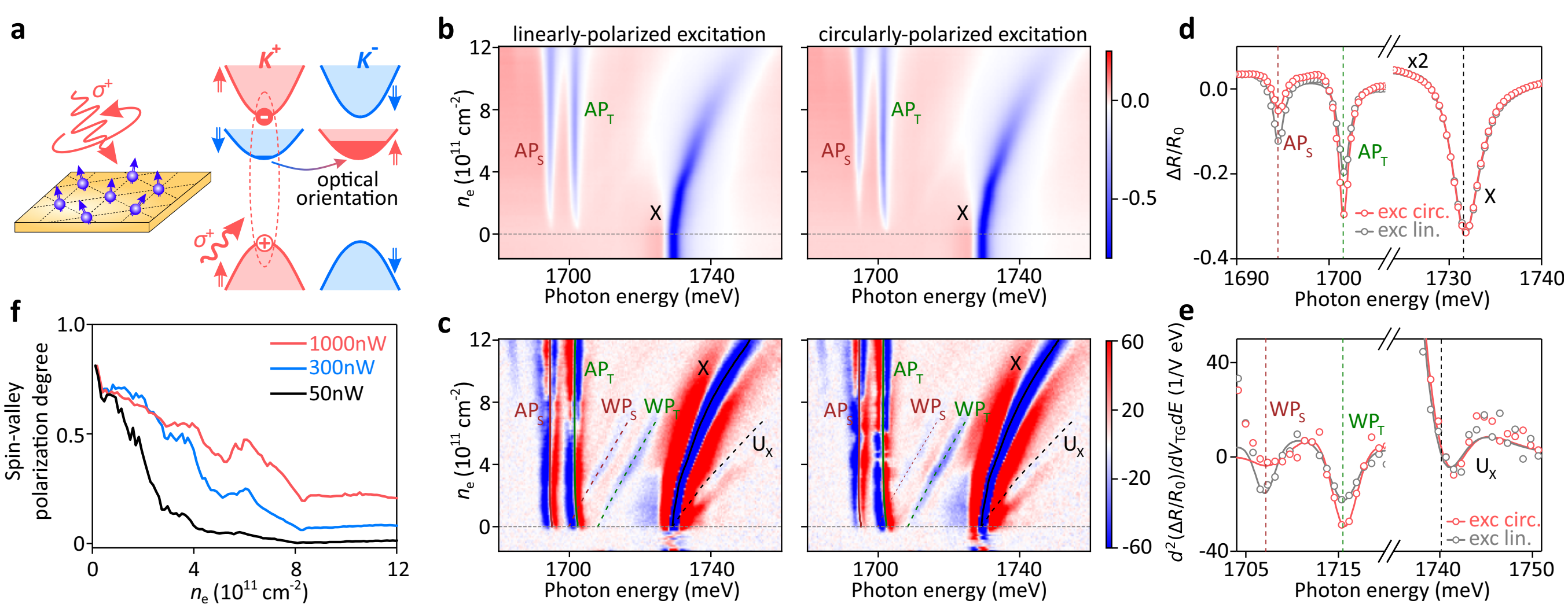

Figure S2. **Optical spin orientation of the zero-field WC.** (**a**) Cartoon illustrating the idea of WC spin pumping under circularly-polarized optical excitation. (**b**,**c**) Electron-density evolutions of bare (**b**) and differentiated (**c**) reflectance contrast spectra measured at $B = 0$ and $T = 1.6$ K under co-linearly (left) and co-circularly (right) polarized optical excitation with power $P = 300$ nW and a broadband ≈ 120-meV-wide spectrum centered around the X resonance. To suppress the noise, a spectrally flat n_e-dependent background was subtracted from the data in (**b**). (**d**,**e**) Linecuts through the maps in (**b**,**c**) at $n_e \approx 3 \cdot 10^{11}$ cm^{-2} showing the main resonances (**d**) and the corresponding WP/umklapp transitions (**e**) under two excitation polarization settings (same as Figs. 4**i,j** from the main text). The data points in (**e**) were binned along the energy axis. Solid lines mark the fitted spectral profiles of the visible resonances. (**f**) Spin-valley polarization degree of the WC under circularly-polarized excitation of different powers (as indicated), determined as $\langle S_z \rangle = (f^{\rm circ}_{\rm AP_T}/f^{\rm lin}_{\rm AP_T} - f^{\rm circ}_{\rm AP_S}/f^{\rm lin}_{\rm AP_S})/(f^{\rm circ}_{\rm AP_T}/f^{\rm lin}_{\rm AP_T} + f^{\rm circ}_{\rm AP_S}/f^{\rm lin}_{\rm AP_S})$, where $\rm AP^{circ}_{T,S}$ ($\rm AP^{lin}_{T,S}$) denote oscillator strengths of the $AP_{T,S}$ resonances obtained from TM fitting of their spectral profiles in circularly (linearly) excited reflectance spectra.

S3. DATA FOR THE DEVICE B

The results presented in the main text were reproduced in another sample (device B), which was fabricated without a top capping hBN layer (Fig. S3**a**). In this device, the thicknesses of the top and bottom hBN encapsulation layers were chosen to ensure destructive interference between the resonant light reflected from various interfaces away from the WSe_2 monolayer. As a consequence, the background reflectance R_0 of this device is drastically smaller than that of device A from the main text, resulting in sizably larger reflectance contrast values $\Delta R/R_0$ of optical resonances in device B. For the same reason, these resonances, while still exhibiting a close-to-Lorentzian lineshape, appear as peaks rather than dips, in contrast to device A.

Low-temperature spectroscopy measurements of device B are summarized in Figs. S3**b-e**. Consistent with the main text, we observe prominent signatures of exciton-umklapp transitions as well as both singlet and triplet WP resonances in the low-density regime, where the electrons form a WC. The energy splittings between each of these resonances and their main counterparts (i.e., X or $\mathrm{AP_S}/\mathrm{AP_T}$) exhibit the same behavior as for device A. In particular, their increase with n_e can be described by linear relations of the same slope but with finite offsets in the case of WPs.

We also investigated thermal melting of the WC in device B. Fig. S4 shows the corresponding temperature-dependent results, presented in a similar fashion as in Fig. 2 of the main text. Both $\mathrm{U_X}$ and $\mathrm{WP_{S,T}}$ resonances reveal similar dependence on T and n_e, exhibiting a characteristic dome-shaped phase diagram associated with the WC melting.

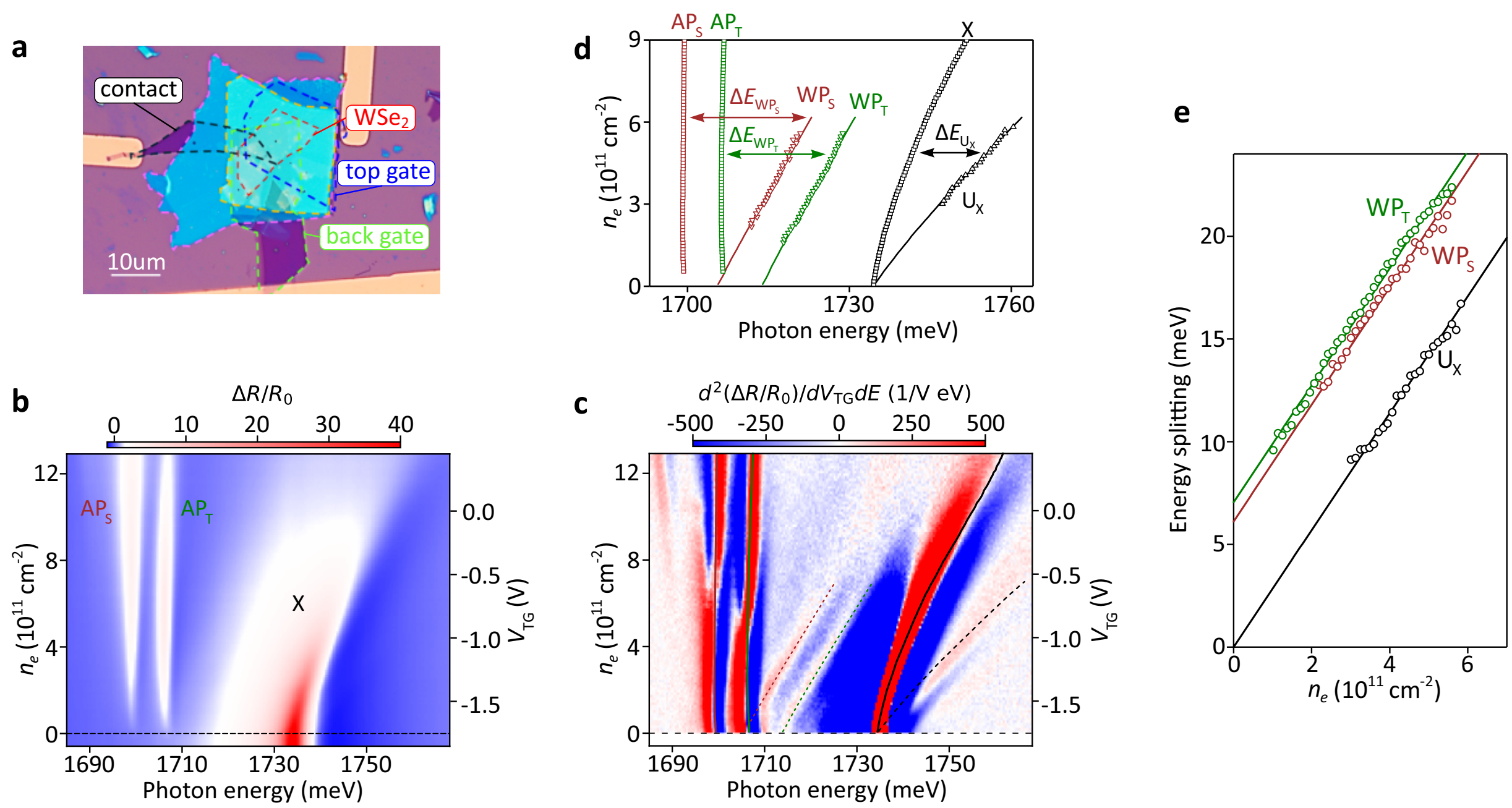

Figure S3. **Wigner crystal polaron spectroscopy in device B.** (**a**) Optical micrograph of device B. (**b, c**) Gate-voltage evolution of the reflectance contrast spectra (**b**) and their derivative with respect to gate voltage and photon energy (**c**) at $B = 0$ and $T = 1.6\,\mathrm{K}$, with resonances marked in the same manner as for device A described in the main text. (**d**) Extracted energies of the X, $\mathrm{AP_T}$, $\mathrm{AP_S}$, $\mathrm{U_X}$, $\mathrm{WP_T}$, and $\mathrm{WP_S}$ resonances at low density. (**e**) Density evolution of the energy splittings between the main resonances and their corresponding umklapps. The dependencies are fitted using the same procedure as for the device A, yielding $m_\mathrm{X} \approx 0.61\,m_0$, $\Delta_{\mathrm{U_X}} = 0$, $\Delta_{\mathrm{WP_S}} \approx 6\,\mathrm{meV}$, and $\Delta_{\mathrm{WP_T}} \approx 7\,\mathrm{meV}$.

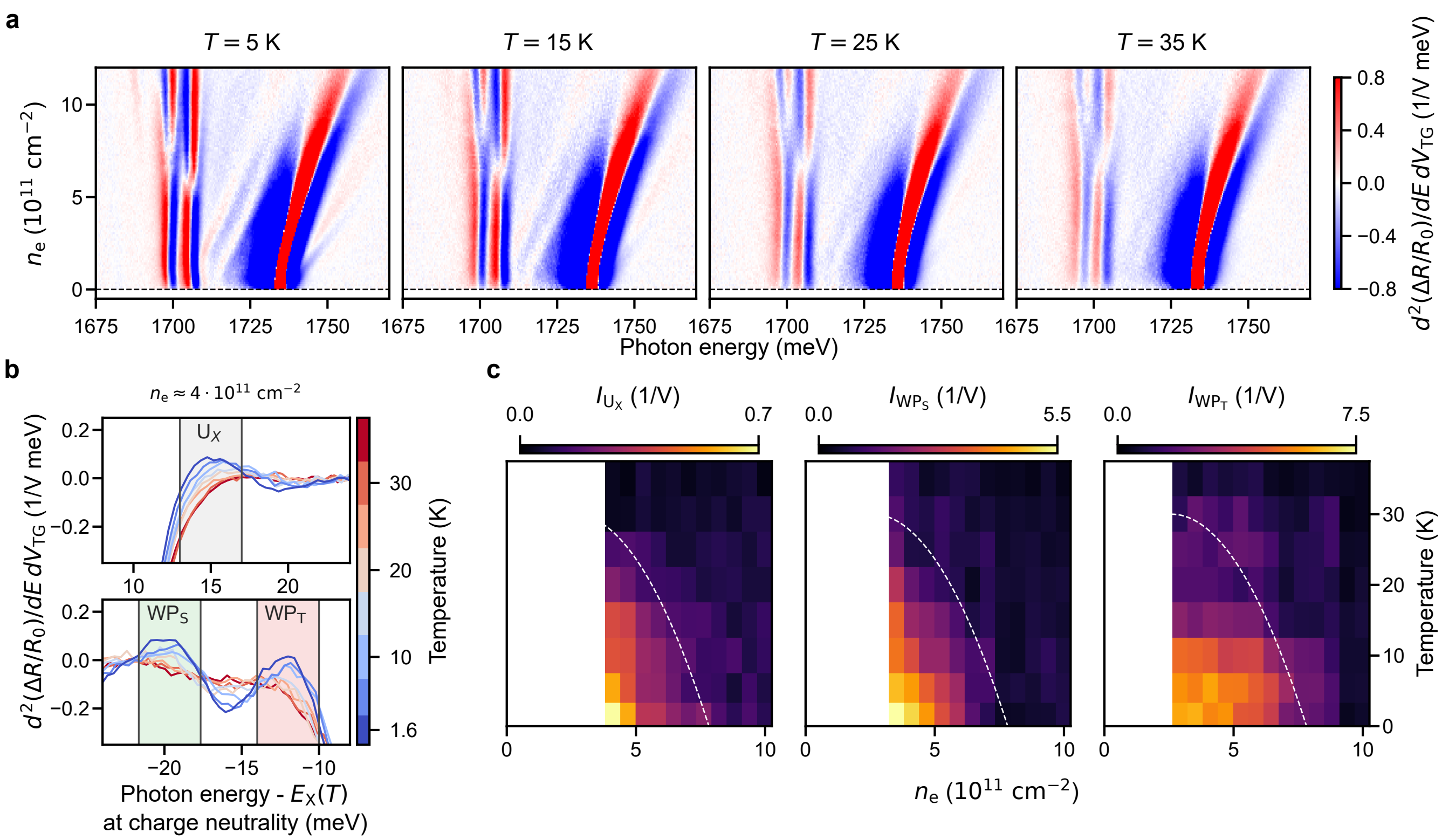

Figure S4. **Temperature and density evolution of umklapp and WP resonances in device B.** (**a**) Representative differentiated reflectance contrast spectra of device B at $T = 5\,\mathrm{K}$, $15\,\mathrm{K}$, $25\,\mathrm{K}$ and $35\,\mathrm{K}$. (**b**) Energy-aligned linecuts at $n_\mathrm{e} \approx 4 \cdot 10^{11}$ cm^{-2} through the differentiated spectra in **a**, taken at $T = 1.6\,\mathrm{K}$–$35\,\mathrm{K}$. Shaded regions indicate the energy windows used to evaluate the spectral weights of the respective resonances. (**c**) Temperature–density phase diagrams of the Wigner crystal extracted based on $\mathrm{U_X}$ (left), $\mathrm{WP_S}$ (middle), and $\mathrm{WP_T}$ (right) using the same procedure as for the main device.

S4. THEORY: POLARON FORMATION

To understand the formation of the Wigner crystal polaron, we consider the following model system:

$$
\begin{aligned}
H_\mathrm{X} &= \sum_{\mathbf{k}} \epsilon^\mathrm{X}_{\mathbf{k}} x^\dagger_{\mathbf{k}} x_{\mathbf{k}}, \\
H_\mathrm{e} &= \sum_{\sigma\mathbf{k}} \epsilon^\mathrm{e}_{\mathbf{k}} c^\dagger_{\sigma\mathbf{k}} c_{\sigma\mathbf{k}} + \frac{1}{N} \sum_{\substack{\mathbf{k},\mathbf{k}',\mathbf{q} \\ \sigma\sigma'}} V^{\mathrm{e-e}}_{\mathbf{q}} c^\dagger_{\sigma\mathbf{k}+\mathbf{q}} c^\dagger_{\sigma'\mathbf{k}'-\mathbf{q}} c_{\sigma'\mathbf{k}'} c_{\sigma\mathbf{k}}, \\
H_\mathrm{X-e} &= \frac{1}{N} \sum_{\substack{\mathbf{k},\mathbf{k}',\mathbf{q} \\ \sigma}} U^{\mathrm{X-e}}_{\sigma} c^\dagger_{\sigma\mathbf{k}+\mathbf{q}} c_{\sigma\mathbf{k}} x^\dagger_{\mathbf{k}'-\mathbf{q}} x_{\mathbf{k}'},
\end{aligned}
\tag{S1}
$$

with $\epsilon^\mathrm{X}_{\mathbf{k}} = \hbar^2|\mathbf{k}|^2/(2m_\mathrm{X})$ and $\epsilon^\mathrm{e}_{\mathbf{k}} = \hbar^2|\mathbf{k}|^2/(2m^*_\mathrm{b,e})$. The electron-exciton interaction $U^{\mathrm{X-e}}_\sigma$ is taken to be attractive and contact-like. We treat the exciton as a tightly-bound impurity, which is immersed in an electronic state. The two electron flavors $\sigma \in \{K^\pm\}$ correspond to the bottom conduction band in the $K^\pm$ valley. Under the assumption that repulsive Coulomb interactions $V^{\mathrm{e-e}}_{\mathbf{q}}$ stabilize the WC, we perform a mean-field decoupling of the electron interactions. The resulting mean-field Hamiltonian for the electrons consequently includes a periodic potential $V^\mathrm{e}(\mathbf{r}) \propto \langle n_\mathrm{e}(\mathbf{r})\rangle$, whose strength scales linearly with the electron density. In other words, we model the Wigner crystal as a spontaneously stabilized charge-density wave composed of mobile electrons subject to a mean-field periodic potential $V^\mathrm{e}(\mathbf{r})$ generated by the remaining electrons. Concretely, we parameterize the strength of this periodic potential as $V^\mathrm{e} = \Delta_\mathrm{WC}$, where the WC gap Δ_WC depends on the strength of electron-electron interactions. Since V^e scales linearly with density n_e, we take the ratio $\Delta_\mathrm{WC}/E_\mathrm{F}$ to be constant in density, with the Fermi energy $E_\mathrm{F} = \pi\hbar^2 n_\mathrm{e}/m^*_\mathrm{b,e}$. We use Δ_WC as a tuning parameter in our calculation. The triangular lattice constant of the WC is also related to the

electron density $a_{\rm WC}^2 = 2/(\sqrt{3}n_{\rm e})$. We solve the electronic mean-field Hamiltonian by a change to its eigenbasis

$$H_{\rm e} = \sum_{\sigma,\mathbf{k},\lambda} \varepsilon_{\mathbf{k}}^{\lambda} \gamma^{\dagger}_{\sigma\lambda\mathbf{k}} \gamma_{\sigma\lambda\mathbf{k}} \quad \text{with} \quad \gamma^{\dagger}_{\sigma\lambda\mathbf{k}} = \sum_{\mathbf{G}} \mathcal{U}_{\lambda\mathbf{G}}(\mathbf{k}) c^{\dagger}_{\sigma\mathbf{k}+\mathbf{G}}, \tag{S2}$$

where $\mathbf{k}$ is now defined within the first Brillouin zone of the Wigner crystal, $\mathbf{G}$ are its reciprocal lattice vectors, and λ are mean-field band indices. In this basis, the electron-exciton interactions become

$$H_{\rm X-e} = \frac{1}{N} \sum_{\lambda\mu,\sigma} \sum_{\mathbf{G},\mathbf{G}'} \sum_{\mathbf{k},\mathbf{k}',\mathbf{q}\in BZ} [U_{\sigma}^{\rm X-e} \Lambda^{\lambda\mu}_{\mathbf{G}\mathbf{G}'} \times \gamma^{\dagger}_{\sigma\mu\mathbf{k}} \gamma_{\sigma\lambda\mathbf{p}} x^{\dagger}_{\mathbf{q}-\mathbf{k}+\mathbf{p}+\mathbf{G}} x_{\mathbf{q}+\mathbf{G}'}], \tag{S3}$$

where the form factors

$$\Lambda^{\lambda\mu}_{\mathbf{G}\mathbf{G}'}(\mathbf{k},\mathbf{k}') = \frac{1}{N} \sum_{\mathbf{Q}} \mathcal{U}^{*}_{\lambda\mathbf{Q}-\mathbf{G}}(\mathbf{k}) \mathcal{U}_{\mu\mathbf{Q}-\mathbf{G}'}(\mathbf{k}'), \tag{S4}$$

mediate interactions between excitons and the WC quasiparticles $\gamma^{\dagger}$. In particular, these exciton-electron interactions allow for scattering processes of excitons with different reciprocal lattice vectors $\mathbf{G}$. To describe the formation of the WC polaron, we employ the Chevy approximation [S11], which was previously used to describe polaron formation in the presence of charge ordering [S12, S13] and has been shown to yield consistent results with exact diagonalization [S14]. Concretely, we make a variational ansatz for the many-body wave function

$$|\Psi_{\mathbf{p}}\rangle = \sum_{\mathbf{G}} \varphi_{\mathbf{G}}(\mathbf{p}) x^{\dagger}_{\mathbf{p}+\mathbf{G}} |{\rm GS}\rangle + \sum_{\mathbf{G},\sigma} \sum_{\mathbf{k},\mathbf{q},\alpha,\beta} \phi^{\mathbf{k}\mathbf{q}\alpha\beta}_{\mathbf{G},\sigma} x^{\dagger}_{\mathbf{p}+\mathbf{q}-\mathbf{k}+\mathbf{G}} \gamma^{\dagger}_{\sigma\alpha\mathbf{k}} \gamma_{\sigma\beta\mathbf{q}} |{\rm GS}\rangle \tag{S5}$$

restricted to the subspace of the exciton interacting with a single particle-hole excitation of the WC. Here $|{\rm GS}\rangle$ is the electronic ground state. By projecting the full Hamiltonian onto the Chevy-ansatz, we find the following equations:

$$\sum_{\mathbf{G}'} \left[\epsilon^{\rm X}_{\mathbf{p}+\mathbf{G}} \delta_{\mathbf{G},\mathbf{G}'} + V^{\rm X}_{\mathbf{G}-\mathbf{G}'} + \sum_{\mathbf{q}\beta,\sigma} U_{\sigma}^{\rm X-e} \Lambda^{\beta\beta}_{\mathbf{G}\mathbf{G}'}(\mathbf{q},\mathbf{q})\right] \varphi_{\mathbf{G}'}(\mathbf{p}) + \sum_{\mathbf{G}'\sigma} \sum_{\mathbf{q}\beta,\mathbf{k}\alpha} U_{\sigma}^{\rm X-e} \Lambda^{\beta\alpha}_{\mathbf{G}\mathbf{G}'}(\mathbf{q},\mathbf{k}) \phi^{\mathbf{k}\mathbf{q}\alpha\beta}_{\mathbf{G}',\sigma}(\mathbf{p}) = E_{\mathbf{p}} \varphi_{\mathbf{G}}(\mathbf{p}) \tag{S6}$$

and

$$\sum_{\mathbf{G}'} \left[(\epsilon^{\rm X}_{\mathbf{p}+\mathbf{q}-\mathbf{k}+\mathbf{G}} + \varepsilon^{\alpha}_{\mathbf{k}} - \varepsilon^{\beta}_{\mathbf{q}}) \delta_{\mathbf{G},\mathbf{G}'} + V^{\rm X}_{\mathbf{G}-\mathbf{G}'} + \sum_{\mathbf{q}'\beta',\sigma'} U_{\sigma'}^{\rm X-e} \Lambda^{\beta'\beta'}_{\mathbf{G}\mathbf{G}'}(\mathbf{q}',\mathbf{q}')\right] \phi^{\mathbf{k}\mathbf{q}\alpha\beta}_{\mathbf{G}',\sigma}(\mathbf{p}) + \sum_{\mathbf{G}'} U_{\sigma}^{\rm X-e} \Lambda^{\alpha\beta}_{\mathbf{G}\mathbf{G}'}(\mathbf{k},\mathbf{q}) \varphi_{\mathbf{G}'}(\mathbf{p}) + $$
$$+ \sum_{\mathbf{G}'\mathbf{k}'\alpha'} U_{\sigma}^{\rm X-e} \Lambda^{\alpha\alpha'}_{\mathbf{G}\mathbf{G}'}(\mathbf{k},\mathbf{k}') \phi^{\mathbf{k}'\mathbf{q}\alpha'\beta}_{\mathbf{G}',\sigma}(\mathbf{p}) - \sum_{\mathbf{G}'\mathbf{q}'\beta'} U_{\sigma}^{\rm X-e} \Lambda^{\beta'\beta}_{\mathbf{G}\mathbf{G}'}(\mathbf{q}',\mathbf{q}) \phi^{\mathbf{k}\mathbf{q}'\alpha\beta'}_{\mathbf{G}',\sigma}(\mathbf{p}) = E_{\mathbf{p}} \phi^{\mathbf{k}\mathbf{q}\alpha\beta}_{\mathbf{G},\sigma}(\mathbf{p}), \tag{S7}$$

which we solve numerically to obtain the wave function $|\Psi_{\mathbf{p}}\rangle$. Here, we have also explicitly included a periodic potential $V^{\rm X}$ for the excitons, which they experience due to the formation of the WC. For the Chevy calculations used to generate Fig. 3**c-f** we include $N_{\rm bands} = 3$ bands, and $N_{\mathbf{G}} = 3 \times 3$ reciprocal lattice vectors, centered around $\mathbf{G} = 0$. The momentum sums over the first Brillouin zone contain $N = 16$ momenta. We use an exciton mass $m_{\rm X} = 0.7 m_0$ and an electron mass $m^{*}_{\rm b,e} = 0.45 m_0$ [S15, S16]. We have checked that our qualitative conclusions do not depend on the precise value of these microscopic parameters. For the results presented in Figs. 3**c-e** in the main text, we fix the electron density to $n_{\rm e} = 5 \cdot 10^{11}$ cm^{-2}. We determine the electron-exciton interaction strength $|U^{\rm X-e}|$ based on the splitting between AP and X, which we fix to 33 meV, roughly corresponding to the experimental value for the singlet $\text{AP}_{\rm S}$ in the zero density limit. From bottom panel to top panel in Figs. 3**c-e**, the electron and exciton potential are $V^{\rm e} = 0$, $V^{\rm X} = 0$ (**e**), $V^{\rm e} = 0.01 E_{\rm F}$, $V^{\rm X} = 0.3 E_{\rm F}$ (**d**), $V^{\rm e} = 1.80 E_{\rm F}$, $V^{\rm X} = 0.3 E_{\rm F}$ (**c**). The respective values are representative examples for the three regimes of Wigner crystal strength discussed in the main text. The three panels shown in Figs. 3**c-e** should be understood as illustrative examples in the three regimes and not as quantitative predictions. For all periodic potentials, we assume a simple harmonic form, where $V_{\mathbf{G}} =$ const. for the first shell of reciprocal lattice vectors and zero otherwise.

To make comparisons with the optically measured spectra, we calculate the zero-momentum exciton spectral function

$$\mathcal{A}_{\rm X}(\mathbf{p}=0,\omega) = \sum_{n} |\langle \Psi_{n,\mathbf{p}} | x^{\dagger}_{\mathbf{p}} | 0 \rangle|^2 \delta(\omega - E_{n,\mathbf{p}}), \tag{S8}$$

where the sum runs over all eigenstates n of Eqs. (S6), (S7). To simplify the analysis of the theoretical model, we assume spin-independent electron-exciton interactions, which result in a single AP resonance. As expected, we find that the splitting between the exciton and its umklapp peak predominantly depends on the exciton mass m_{X} and the electron density n_{e}, and is not sensitive to the WC gap Δ_{WC} and the electron-exciton interaction $U^{\mathrm{X-e}}$. By contrast, the splitting between the AP and the WP sensitively depends on both the WC gap Δ_{WC} and the electron-exciton interaction $U^{\mathrm{X-e}}$. Furthermore, the oscillator strength of the exciton umklapp peak depends on the strength of the potential experienced by the excitons V^{X}, while the oscillator strength of the WP predominantly depends on V^{e}, implying that dressing due to electron-exciton interactions plays a dominant role.

We emphasize that neither the AP nor the WP should be understood as a simple bound state between an exciton and another electron (i.e., a trion). Instead, both AP and WP are excitons dressed by collective excitations of an underlying electronic state, which in our case is a Wigner crystal. The "bare" AP corresponds to a dressed zero-momentum exciton, while the "bare" WP is a dressed higher-momentum exciton with $k \approx k_{\mathrm{W}}$. Strong electron-exciton interactions then lead to a hybridization between the "bare" polarons, as explained below. We note that, strictly speaking, since the AP itself is dressed by collective excitations of the Wigner crystal, one could already call it a "Wigner crystal polaron." But since it is continuously connected to the conventional AP in the high-density regime (where the WC ceases to exist), we still refer to it as an attractive polaron. This is to distinguish it from the WP, which only exists inside the WC regime.

We would also like to comment on independent theoretical [S17] and experimental [S18] works, which present an alternative perspective of the Wigner polaron excitations as arising from dressing of excitons by phonons of the WC. Although predicting a slightly different density evolution of the WP–AP energy splitting, $\Delta E_{\mathrm{WP}} \propto n_{\mathrm{e}}^{3/4}$, this theoretical framework is complementary to ours, and approaches the mathematically unsolvable many-body problem of WP formation from an opposite extreme. Specifically, instead of treating the WC as a charge-density wave consisting of mobile electrons subject to a mean-field periodic potential (which is the approach presented in our work), the WC phonon model describes WC electrons as distinguishable particles with non-overlapping wave functions. In this limit, the exciton forming a trion at a given WC site locally distorts the WC lattice and thus becomes dressed into a WP by collective neutral WC phonon modes. Consequently, this approach is expected to better capture WC properties in the low-density regime, where WC electrons are strongly localized. Since this regime is also more strongly affected by disorder, understanding the crossover between these two approximate descriptions of Wigner polarons will likely require incorporating more realistic excitation spectra of disorder-pinned Wigner solids, possibly through systematic studies of samples with varying disorder levels.

S5. THEORY: HYBRIDIZATION MODEL

The experimentally observed splitting of the Wigner crystal polaron ΔE_{WP} from the AP can be explained by a simple effective model in which electron-exciton interactions induce hybridization between "bare" AP and WP states corresponding to polarons formed from excitons with $k \approx 0$ and $k \approx k_{\mathrm{W}}$. Because exciton-electron interactions couple exciton states with different momenta, these "bare" states are not eigenstates of the many-body Hamiltonian, which is the fundamental reason why they hybridize.

The above model is given by Eq. (1) of the main text, which is reproduced here:

$$H_{\mathrm{hyb}} = \begin{pmatrix} E_{\mathrm{AP}} & V_{\mathrm{hyb}}(n_{\mathrm{e}}) \\ V^{*}_{\mathrm{hyb}}(n_{\mathrm{e}}) & E_{\mathrm{AP}} + \frac{h^2}{\sqrt{3}}\frac{n_{\mathrm{e}}}{m_{\mathrm{X}}} \end{pmatrix}.$$

The hybridization strength V_{hyb} depends both on the effective contact interaction $U^{\mathrm{X-e}}_{\mathrm{eff}}$ and the WC gap Δ_{WC}, which enters in the electron-exciton interactions through the form factors $\Lambda^{\lambda,\mu}_{\mathbf{G},\mathbf{G}'}$ defined by Eq. (S4). We find that these form factors $\Lambda^{\lambda,\mu}_{\mathbf{G},\mathbf{G}'}$ increase with the WC gap, explaining why the WP exhibits larger spectral weight for a more stable WC. Keeping the number of mean-field bands included in our calculation fixed implies that the form factors are density independent, so that the only density dependence of the hybridization strength V_{hyb} stems from $U^{\mathrm{X-e}}_{\mathrm{eff}}$. The effective interactions $U^{\mathrm{X-e}}_{\mathrm{eff}}$ can be estimated by the T-matrix that has a logarithmic divergence at low densities in two-dimensional systems $\propto 1/\log(n_0/n_{\mathrm{e}})$ [S19], where $n_0 = m^{*}_{\mathrm{b,e}} E_{\mathrm{b}}/(\pi\hbar^2)$ is the typical density associated with the trion binding energy E_{b}. Within this hybridization model, the splitting between the AP and the WP is given by

$$\Delta E_{\mathrm{WP}} = \sqrt{\left(\frac{h^2}{\sqrt{3}}\frac{n_{\mathrm{e}}}{m_{\mathrm{X}}}\right)^2 + 4|V_{\mathrm{hyb}}(n_{\mathrm{e}})|^2}. \tag{S9}$$

Parameterizing the hybridization as $|V_{\mathrm{hyb}}(n_{\mathrm{e}})|^2 = |V^0_{\mathrm{hyb}}/\log(n_0/n_{\mathrm{e}})|^2$, describes the density dependence of the splittings $\Delta E_{\mathrm{WP_{S,T}}}$ for both Wigner crystal polarons with a single parameter V^0_{hyb}. We use the data for $\mathrm{WP_S}$ to extract

$V^0_{\rm hyb} = 16.4$ meV. This value is then used to predict the density dependence of $\mathrm{WP_T}$, in good agreement with the experimental data (see Fig. 3**g**). To calculate $n_0 = m^*_{\rm b,e}E_{\rm b}/(\pi\hbar^2)$, the respective experimental values of zero-density binding energies of $\mathrm{AP_S}$ and $\mathrm{AP_T}$ are used. Note that in our model we assume $\Delta_{\rm WC}/E_{\rm F}$ to be independent of the electron density, which is a good approximation deep within the WC phase. As the density approaches the critical value n_c, where the WC melts, $\Delta_{\rm WC}/E_{\rm F}$ decreases. This additional density dependence of $V_{\rm hyb}(n_{\rm e})$, arising from the quantum melting of the WC, is not captured in our simple model. Nevertheless, even at high densities, the predictions of the model remain in good agreement with the experimental data. This is because, in this regime, the dominant contribution to the splitting $\Delta E_{\rm WP}$ originates from the diagonal Bragg-scattering term proportional to $n_{\rm e}/m_{\rm X}$, which is independent of $\Delta_{\rm WC}$. Note that the mass determining the slope of $\Delta E_{\rm WP}$ increase with $n_{\rm e}$ corresponds to the translational mass of the bare exciton $m_{\rm X}$, which is consistent with the fact that WP can be understood as a Bragg-scattered exciton dressed by collective excitations of the Wigner crystal.

S6. THEORY: MAGNETIC FIELD DEPENDENCE

To model the Zeeman splittings of the optical resonances involving umklapp-scattered excitons, we follow the approach of Ref. [S20, S21]. Concretely, we calculate the magnetic-field-induced splitting of an umklapp-scattered exciton as a function of the sign and strength of the electron-exciton interactions. Due to electron-hole exchange interactions, the K^+ and K^- valley excitons (which at zero momentum couple to σ^+ and σ^- polarized light, respectively) are coupled at finite momentum [S20, S22, S23]. In the spin-valley basis $\mathbf{x}^\dagger_{\mathbf{k}} = (x^\dagger_{\mathbf{k},+},\ x^\dagger_{\mathbf{k},-})$, the excitons are described by the Hamiltonian $H = H_0 + H_{\rm pot} + H_{\rm z}$, with

$$\begin{aligned} H_0 &= \sum_{\mathbf{k}} \mathbf{x}^\dagger_{\mathbf{k}} \left[\frac{\hbar^2\mathbf{k}^2}{2m_{\rm X}} + J\frac{|\mathbf{k}|}{K} \begin{pmatrix} 1 & e^{-2i\theta_{\mathbf{k}}} \\ e^{2i\theta_{\mathbf{k}}} & 1 \end{pmatrix} \right] \mathbf{x}_{\mathbf{k}}, \\ H_{\rm pot} &= \sum_{\mathbf{k}\in \mathrm{BZ}} \sum_{\mathbf{G},\mathbf{G}'} \mathbf{x}^\dagger_{\mathbf{k}+\mathbf{G}} V^{\rm X}_{\mathbf{G}-\mathbf{G}'} \mathbf{x}_{\mathbf{k}+\mathbf{G}'}, \\ H_{\rm z} &= \frac{g\mu_B B}{2} \sum_{\mathbf{k}} \mathbf{x}^\dagger_{\mathbf{k}} \sigma^z \mathbf{x}_{\mathbf{k}}. \end{aligned} \tag{S10}$$

The first term H_0 describes the kinetic energy of the excitons and their coupling due to electron-hole exchange interactions, parameterized by J. Here, $K = 4\pi/(3a_0)$ is the momentum of the $K^\pm$ valley, $a_0 \approx 0.3$ nm is the lattice constant of $\mathrm{WSe_2}$, and $\theta_{\mathbf{k}} = \arctan(k_y/k_x)$. The second term $H_{\rm pot}$ models the periodic potential felt by the excitons due to the presence of a Wigner crystal, which in this approximation is treated as a static background. The potential $V^{\rm X}$ originates from a mean-field decoupling of electron-exciton interactions. Consequently, a positive (negative) potential $V^{\rm X}$ corresponds to repulsive (attractive) interactions. Finally, $H_{\rm z}$ captures the bare Zeeman splitting due to a finite magnetic field B. We assume a g-factor of $g = 4$.

To obtain the oscillator strength with respect to $\sigma^\pm$ polarized light of a given state, we compute its overlap with the zero-momentum state $|\mathbf{k} = 0, \pm\rangle = x^\dagger_{\mathbf{k}=0,\pm}|0\rangle$ (see Fig. S5**a**,**b**). We then calculate the Zeeman splitting for the optically active umklapp peak. As expected, for weak interactions, the Zeeman splitting of umklapp-scattered excitons is very small [S8, S20]. However, we find that the magnitude of the Zeeman umklapp splitting increases with stronger exciton-electron interaction potential $|V^{\rm X}|$ and decreases for larger electron-hole exchange J (see Fig. S5**c**). Most importantly, our calculations reveal that attractive electron-exciton interactions ($V^{\rm X} < 0$) lead to a positive Zeeman umklapp splitting, while repulsive interactions ($V^{\rm X} > 0$) lead to a negative one. This is consistent with the experimental observation from the main text that the Zeeman splitting for the X, which is a repulsive polaron, and its umklapp are opposite in sign. On the other hand, the AP and WP, which are dominated by attractive electron-exciton interactions, exhibit Zeeman splittings of the same sign. We emphasize that our magnetic-field calculations include electron-exciton interactions only on a mean-field level, which is not sufficient to capture the AP and WP formation. As such, we do not expect to quantitatively describe the Zeeman splitting of the WP and its complex dependency as a function of density $n_{\rm e}$. Nevertheless, we qualitatively explain the sign of the Zeeman splitting for the WP. Understanding the details of exciton-polaron formation in a magnetic field is an interesting question for future theoretical work.

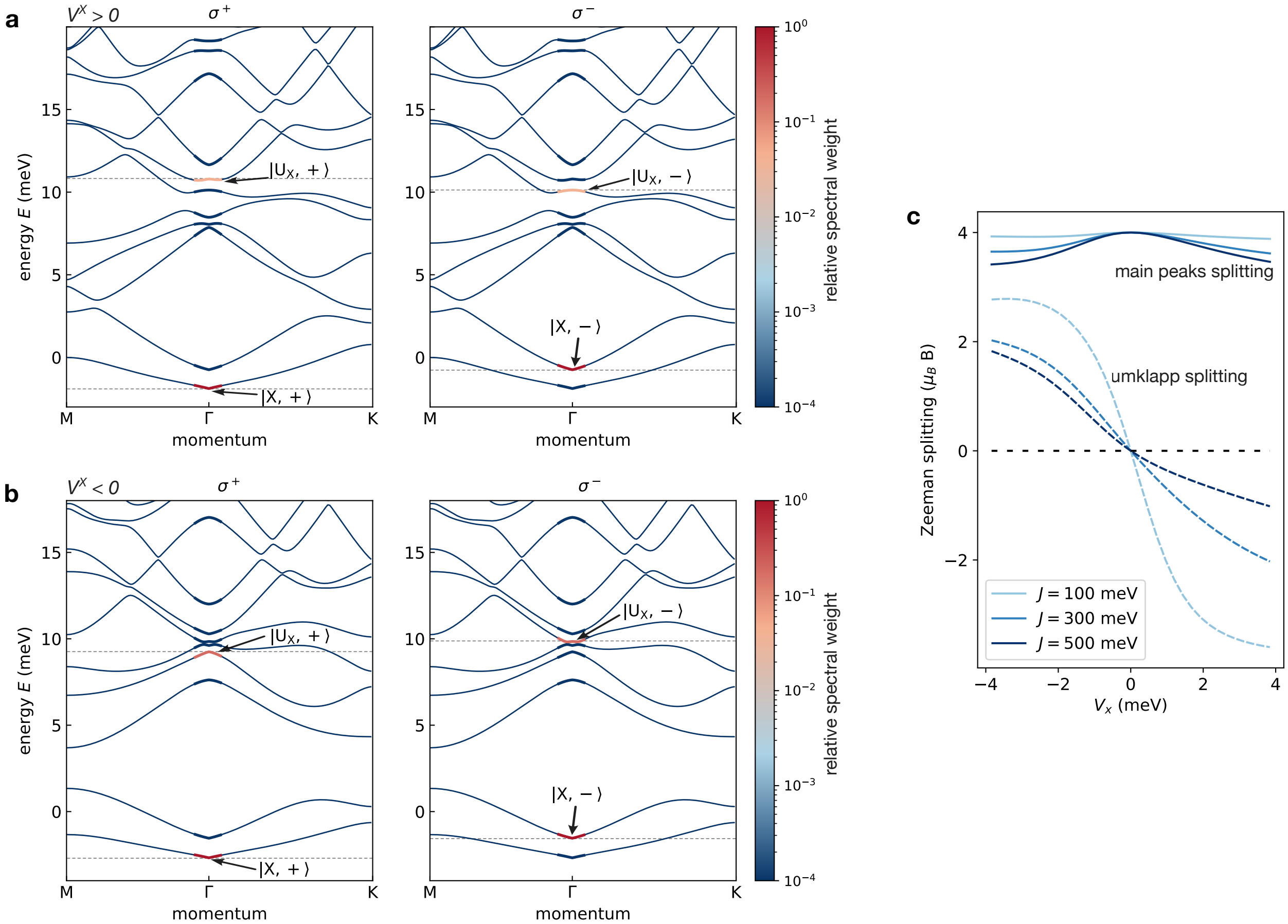

Figure S5. **Interaction-dependent Zeeman splitting of optical resonances involving exciton-umklapp scattering.** Exciton bands for repulsive (**a**) and attractive (**b**) electron-exciton interactions in the presence of a periodically-ordered WC. The spectral weight of each state (relative to the strongest peak) is indicated through the color scale. Left (Right): coupling to σ^- (σ^+) polarized light. We used $J = 150$ meV and $|V^{\mathrm{X}}| = 2$ meV at $B = 5$ T and $n_{\mathrm{e}} = 4 \cdot 10^{11}$ cm^{-2}. (**c**) Zeeman splitting between the main peaks $|\mathrm{X}, \pm\rangle$ (solid lines) and between the umklapp peaks $|\mathrm{U_X}, \pm\rangle$ (dashed lines) for various J at $B = 5$ T as a function of the exciton potential strength V^{X}. Negative V^{X}, corresponding to attractive electron-exciton interactions, leads to positive Zeeman splitting for the umklapp peaks. Conversely, positive V^{X} (repulsive electron-exciton interactions) imply negative Zeeman splitting for the umklapp peaks.

S7. THEORY: DISCUSSION OF SINGLET AND TRIPLET AP

In Sec. S4, we analyzed polaron formation in the presence of a Wigner crystal, demonstrating how an AP and the associated WP form. So far, our discussion has focused on a single AP branch and its corresponding WP replica. However, experimentally, two distinct resonances are observed for electron-doped WSe_2: the singlet and triplet attractive polaron ($\mathrm{AP_S}$ and $\mathrm{AP_T}$) and their associated Wigner crystal polarons ($\mathrm{WP_S}$ and $\mathrm{WP_T}$). As discussed in the main text, they form due to interactions of the exciton with electrons in the same and opposite valleys, respectively. Here, we discuss the challenges that arise in theoretically modeling the formation of these two distinct AP and WP branches.

To first approximation, the formation of singlet and triplet AP/WP transitions can be treated as independent problems. Within this approximation, the model of Sec. S4 applies to both cases separately, once the experimentally determined AP energies are used as input. Despite its simplicity, this approach already captures the density dependence of the WP splitting ΔE_{WP} for both singlet and triplet, in good agreement with experiment (see Fig. 3**g**), thereby justifying this treatment. The independence of WP formation from the AP spin structure is also consistent with our results on the hole-doped side (see Methods Sec. 9), where only a singlet trion is bound and only one pair of AP and WP transitions appears in the low-density spectra, with their splitting following a similar dependence on the carrier density.

A naive extension of the above simplistic approach is to incorporate spin-dependent electron interactions. In this

approximation, the energy difference between the singlet and triplet AP is modeled by assuming different interaction strengths between excitons and electrons in the same and opposite valleys. However, we find that Chevy-type calculations based on this assumption fail to reproduce the experimental results even for the APs, as both the binding energy and spectral weight of the singlet AP are strongly overestimated. Moreover, this assumption would also imply a large influence of spin disorder on the WP formation in the case where only a singlet trion is bound, which appears inconsistent with our experiments on the hole WC (see Methods Sec. 9). These observations indicate that introducing spin dependence in the exciton-electron interaction alone is insufficient to explain the observed singlet and triplet AP branches. We note that the results of Ref. [S17] suggest that this shortcoming can be overcome when the AP_S and AP_T wave-functions are spatially separated, which restricts hybridization between them.

Importantly, recent work in Ref. [S16] highlights the significant role the internal structure of the exciton has in accurately capturing the formation of the singlet and triplet trions. They argue that the measured energies of AP_S and AP_T can only be explained by taking the non-trivial internal structure of exciton and trion wave functions into account. Such a treatment is beyond our model, where we treat the exciton as a tightly-bound point-like particle, whose wave function has s-wave character. Incorporating the internal structure of the exciton into our calculations is a challenging theoretical task and requires a purely electronic description of the exciton formation [S13], which is an interesting open problem for future work.

While our simplified framework cannot fully account for the singlet-triplet AP splitting, it does capture the formation of the WP, which is the central focus of this work.

[S1] F. I. B. Williams, P. A. Wright, R. G. Clark, E. Y. Andrei, G. Deville, D. C. Glattli, O. Probst, B. Etienne, C. Dorin, C. T. Foxon, and J. J. Harris. *Conduction threshold and pinning frequency of magnetically induced Wigner solid.* *Phys. Rev. Lett.* **66**, 3285–3288 (1991).

[S2] P. D. Ye, L. W. Engel, D. C. Tsui, R. M. Lewis, L. N. Pfeiffer, and K. West. *Correlation Lengths of the Wigner-Crystal Order in a Two-Dimensional Electron System at High Magnetic Fields.* *Phys. Rev. Lett.* **89**, 176802 (2002).

[S3] Y. Zhou, I. Esterlis, and T. Smoleński. *Electronic crystals in layered materials.* *arXiv:2509.21222* (2025).

[S4] Z. Xiang, H. Li, J. Xiao, M. H. Naik, Z. Ge, Z. He, S. Chen, J. Nie, S. Li, Y. Jiang, R. Sailus, R. Banerjee, T. Taniguchi, K. Watanabe, S. Tongay, S. G. Louie, M. F. Crommie, and F. Wang. *Imaging quantum melting in a disordered 2D Wigner solid.* *Science* **388**, 736–740 (2025).

[S5] Z. Ge, C. Smith, Z. He, Y. Yang, Q. Li, Z. Xiang, J. Xiao, W. Zhou, S. Kahn, M. Erdi, R. Banerjee, T. Taniguchi, K. Watanabe, A. Tongay, M. A. Morales, S. Zhang, F. Wang, and M. F. Crommie. *Visualizing the Impact of Quenched Disorder on 2D Electron Wigner Solids.* *arXiv:2510.12009* (2025).

[S6] D. Vu and S. Das Sarma. *Thermal melting of a quantum electron solid in the presence of strong disorder: Anderson localization versus the Wigner crystal.* *Phys. Rev. B* **106**, L121103 (2022).

[S7] A. Babbar, Z.-J. Li, and S. Das Sarma. *Wigner solid or Anderson solid—2D electrons in strong disorder.* *arXiv:2601.03521* (2026).

[S8] T. Smoleński, P. E. Dolgirev, C. Kuhlenkamp, A. Popert, Y. Shimazaki, P. Back, X. Lu, M. Kroner, K. Watanabe, T. Taniguchi, I. Esterlis, E. Demler, and A. Imamoğlu. *Signatures of Wigner crystal of electrons in a monolayer semiconductor.* *Nature* **595**, 53–57 (2021).

[S9] C. Robert, S. Park, F. Cadiz, L. Lombez, L. Ren, H. Tornatzky, A. Rowe, D. Paget, F. Sirotti, M. Yang, D. Van Tuan, T. Taniguchi, B. Urbaszek, K. Watanabe, T. Amand, H. Dery, and X. Marie. *Spin/valley pumping of resident electrons in WSe_2 and WS_2 monolayers.* *Nat. Comm.* **12**, 5455 (2021).

[S10] J. Li, M. Goryca, K. Yumigeta, H. Li, S. Tongay, and S. A. Crooker. *Valley relaxation of resident electrons and holes in a monolayer semiconductor: Dependence on carrier density and the role of substrate-induced disorder.* *Phys. Rev. Mater.* **5**, 044001 (2021).

[S11] F. Chevy. *Universal phase diagram of a strongly interacting Fermi gas with unbalanced spin populations.* *Phys. Rev. A* **74**, 063628 (2006).

[S12] I. Amelio, G. Mazza, and N. Goldman. *Polaron formation in insulators and the key role of hole scattering processes in band insulators, charge density waves, and Mott transitions.* *Phys. Rev. B* **110**, 235302 (2024).

[S13] F. Pichler, M. Hafezi, and M. Knap. *Purely Electronic Model for Exciton-Polaron Formation in Moiré Heterostructures.* *arXiv:2503.07712* (2025).

[S14] I. Amelio and N. Goldman. *Polaron Spectroscopy of Interacting Fermi Systems: Insights from Exact Diagonalization.* *SciPost Phys.* **16**, 056 (2024).

[S15] A. Kormányos, G. Burkard, M. Gmitra, J. Fabian, V. Zólyomi, N. D. Drummond, and V. Fal'ko. *K·p Theory for Two-Dimensional Transition Metal Dichalcogenide Semiconductors.* *2D Mater.* **2**, 022001 (2015).

[S16] A. Christianen and A. Imamoglu. *Asymmetric trions in monolayer transition metal dichalcogenides.* *arXiv:2503.07712* (2025).

[S17] H. S. Adlong, E. Dizer, R. Schmidt, A. Imamoglu, and A. Christianen. *Theory of exciton polarons in 2D Wigner crystals.* *arXiv:2512.16651* (2025).

[S18] L. Zhang, L. Gu, H. S. Adlong, A. Christianen, E. Dizer, R. Ni, R. Ma, S. Park, H. Jang, T. Taniguchi, K. Watanabe,

I. Esterlis, R. Schmidt, A. Imamoglu, and Y. Zhou. *Wigner polarons reveal Wigner crystal dynamics in a monolayer semiconductor.* *arXiv:2512.16631* (2025).

[S19] C. Kuhlenkamp, M. Knap, M. Wagner, R. Schmidt, and A. Imamoğlu. *Tunable Feshbach Resonances and Their Spectral Signatures in Bilayer Semiconductors.* *Phys. Rev. Lett.* **129**, 037401 (2022).

[S20] Y. Shimazaki, C. Kuhlenkamp, I. Schwartz, T. Smoleński, K. Watanabe, T. Taniguchi, M. Kroner, R. Schmidt, M. Knap, and A. Imamoğlu. *Optical Signatures of Periodic Charge Distribution in a Mott-like Correlated Insulator State.* *Phys. Rev. X* **11**, 021027 (2021).

[S21] A. G. Salvador, C. Kuhlenkamp, L. Ciorciaro, M. Knap, and A. Imamoğlu. *Optical Signatures of Periodic Magnetization: The Moiré Zeeman Effect.* *Phys. Rev. Lett.* **128**, 237401 (2022).

[S22] C. R. Zhu, G. Wang, B. L. Liu, X. Marie, X. F. Qiao, X. Zhang, X. X. Wu, H. Fan, P. H. Tan, T. Amand, and B. Urbaszek. *Strain tuning of optical emission energy and polarization in monolayer and bilayer* MoS_2. *Phys. Rev. B* **88**, 121301 (2013).

[S23] H. Yu, G.-B. Liu, P. Gong, X. Xu, and W. Yao. *Dirac cones and Dirac saddle points of bright excitons in monolayer transition metal dichalcogenides.* *Nat. Commun.* **5**, 3876 (2014).
